# EBNA3C facilitates RASSF1A downregulation through ubiquitin-mediated degradation and promoter hypermethylation to drive B-cell proliferation

**DOI:** 10.1371/journal.ppat.1007514

**Published:** 2019-01-07

**Authors:** Shengwei Zhang, Yonggang Pei, Fengchao Lang, Kunfeng Sun, Rajnish Kumar Singh, Zachary L. Lamplugh, Abhik Saha, Erle S. Robertson

**Affiliations:** 1 Department of Otorhinolaryngology-Head and Neck Surgery, and Microbiology, the Tumor Virology Program, Abramson Cancer Center, Perelman School of Medicine at the University of Pennsylvania, Philadelphia, Pennsylvania, United States of America; 2 Department of Life Sciences, Presidency University, Kolkata, India; Brigham and Women’s Hospital, UNITED STATES

## Abstract

EBV latent antigen 3C (EBNA3C) is essential for EBV-induced primary B-cell transformation. Infection by EBV induces hypermethylation of a number of tumor suppressor genes, which contributes to the development of human cancers. The Ras association domain family isoform 1A (RASSF1A) is a cellular tumor suppressor, which regulates a broad range of cellular functions, including apoptosis, cell-cycle arrest, mitotic arrest, and migration. However, the expression of RASSF1A is lost in many human cancers by epigenetic silencing. In the present study, we showed that EBNA3C promoted B-cell transformation by specifically suppressing the expression of RASSF1A. EBNA3C directly interacted with RASSF1A and induced RASSF1A degradation via the ubiquitin-proteasome-dependent pathway. SCF^Skp2^, an E3-ubiquitin ligase, was recruited by EBNA3C to enhance RASSF1A degradation. Moreover, EBNA3C decreased the transcriptional activity of RASSF1A promoter by enhancing its methylation through EBNA3C-mediated modulation of DNMTs expression. EBNA3C also inhibited RASSF1A-mediated cell apoptosis, disrupted RASSF1A-mediated microtubule and chromosomal stability, and promoted cell proliferation by upregulating Cyclin D1 and Cyclin E expression. Our data provides new details, which sheds light on additional mechanisms by which EBNA3C can induce B-cell transformation. This will also facilitate the development of novel therapeutic approaches through targeting of the RASSF1A pathway.

## Author summary

Epstein-Barr virus (EBV) which is associated with multiple lymphoid and epithelial malignancies was the first recognized oncogenic virus in humans. EBNA3C, an essential latent antigen encoded by EBV interacts with numerous host transcription factors and plays an important role in the transformation of primary B-cells. RASSF1A, a tumor suppressor plays a vital role in regulating apoptosis, cell-cycle arrest, and mitotic arrest and is implicated in the development of a number of different cancers. We now demonstrate that EBNA3C can physically interact with RASSF1A and induce RASSF1A degradation through the ubiquitin-proteasome-dependent pathway. Further, the E3 ubiquitin ligase SCF^Skp2^ was recruited by EBNA3C to mediate RASSF1A degradation. Moreover, RASSF1A mRNA expression was also suppressed by EBNA3C. EBNA3C repressed the transcriptional activity of the RASSF1A promoter through induction of its methylation by enhancing DNMT3a expression. EBNA3C regulation of RASSF1A promoted cell proliferation, inhibited RASSF1A-mediated apoptosis and disrupted RASSF1A-mediated microtubule and chromosomal stability. Overall, our results add to our understanding of the many strategies employed by EBNA3C to induce B-cell transformation, which will contribute to new therapeutics for interventions targeting EBV association malignancies.

## Introduction

Epstein-Barr virus (EBV), a double-stranded DNA gammaherpesvirus, was the first recognized and one of the most common oncogenic viruses in humans [1]. It contributes to multiple lymphoid and epithelial malignancies, including Burkitt’s lymphoma (BL), gastric cancer (GC), nasopharyngeal carcinoma (NPC), Hodgkin lymphoma (HL), AIDS-associated B-cell lymphomas, diffuse large B-cell lymphoma (DLBCL), and pyothorax-associated lymphomas [1–4]. B-cell infection by EBV normally results in persistence and latent infection, typically categorized into three major types of latency programs according to different gene expression. During latency III program, commonly established in AIDS-associated B-cell lymphomas, a full set of latency-associated transcripts including nine latent genes along with several small noncoding RNAs and miRNAs are expressed. The latent proteins include six nuclear antigens (EBNA1, EBNA2, EBNA3A, EBNA3B, EBNA3C, and EBNALP) and three viral membrane proteins (LMP1, LMP2A, and LMP2B) [5, 6]. Genetic studies have demonstrated that EBNA2, EBNA3A, EBNA3C, EBNA-LP, and LMP1 are indispensable for the establishment of latency and EBV-mediated transformation of primary B cells [7–10].

EBNA3C (EBV-encoded nuclear antigen 3C), is an important transcriptional regulator with a critical role in viral and cellular gene expression by interacting with numerous host transcription regulators and subsequently modulating their functions [11–13]. Previous studies have shown that a wide range of cellular factors, including but not limited to E2F6 [14], Bcl6 [15], RBP/CSL [16], RBP-Jkappa [17–19], HDAC1 [20], KDM2B [21], p53 [22], IRF-4 [23], and Mdm2 [24, 25] interact with EBNA3C. These interactions disrupt the normal functions of these cellular factors and can drive oncogenic activities. In addition to its transcriptional functions, EBNA3C is also involved in cell-cycle regulation by disrupting multiple cell cycle checkpoints [26] and interacting with Cyclin proteins, including Cyclin D1 [27], Cyclin D2 [28], and Cyclin A [29]. Furthermore, EBNA3C is also important in chromatin reprogramming by recruiting the modifying enzymes histone acetylases and deacetylases [20, 30, 31]. Along with EBNA3A, EBNA3C can also repress several tumor suppressor genes, including p16^INK4a^ [7], p14^ARF^ [7], BCL2L11 [32] and Bim [33] through enhancing H3K27me3 modification at the respective promoter regions [34].

EBV infection of human cells is associated with tumor development, such as nasopharyngeal carcinoma, Burkitt lymphoma, and gastric cancers [35, 36]. Hypo- or hypermethylation-mediated tumor suppressor gene silencing, or oncogene activation, widely contributes to the development many of these human cancers [37]. Previous studies demonstrated that EBV infection can induce extensive DNA methylation to regulate expression of multiple tumor suppressor genes [38]. Previous data from our lab and other groups had identified a number of tumor suppressor genes whose expression were modulated in EBV infected cells by DNA methylation [37, 39]. The Ras association domain family isoform 1A (RASSF1A) is one of these candidates. RASSF1A, a known tumor suppressor gene, plays an essential role in a range of cellular processes by modulating multiple apoptotic and cell cycle checkpoint pathways [40, 41].

RASSF1A localizes with microtubules and promotes their stability [42, 43]. The dynamics of microtubule is affected by directly binding to microtubule-binding protein including Cdc20, to regulate cell migration and mitotic progression [44, 45]. RASSF1A binds to the transcription factor, p120^E4F^ and inhibits the transcription of Cyclin A2, inducing cell cycle arrest in the G1 phase [46]. In addition, RASSF1A also induces G1 arrest by inhibiting the expression levels of Cyclin D1 via the JNK pathway [47]. RASSF1A has also been implicated in the modulation of apoptosis by interacting with multiple pro-apoptotic related proteins, such as MST1 [48], CNK1 [49], MOAP1 [50], and Salvador [51].

Although RASSF1A plays a pivotal role in normal growth control, its expression is lost in many human cancers by promoter hypermethylation [39, 40]. Epigenetic studies with EBV-associated cancers show that EBV infection can be recognized as an epigenetic driver of tumorigenesis due to its ability to induce aberrant promoter methylation of a large number of tumor suppressor genes [37, 38]. During latent infection of EBV, DNA methyltransferase (DNMT) enzymes methylate the EBV genome to repress expression of viral genes. The induced DNA methyltransferases can also suppress cancer-related genes [37, 38, 52]. Three DNA methyltransferase (DNMT) enzymes, DNMT1, DNMT3A, and DNMT3B catalyze methylation of DNA [52]. It was reported that the activity of DNMT1 was increased in EBV-associated gastric cancer and in NPC cell lines, LMP1 up-regulates the expression of these three DNMTs [53]. Furthermore, LMP1 was shown to decrease the expression of RASSF1A by activating intracellular signaling of NF-κB and suppressing the transcriptional activity of the RASSF1A promoter [54]. LMP2A, another EBV-encoded latent nuclear membrane protein, was also shown to upregulate DNMT1 expression and to enhance methylation of the PTEN promoter [55]. Previous studies in our lab showed that EBNA3C not only upregulated the expression of DNMT1 but also interacted with DNMT1 [56]. However, whether EBNA3C or another latent nuclear protein encoded by EBV inhibits expression, and associated functions of RASSF1A by increasing DNMT1 level remains unclear.

Ubiquitin-mediated degradation also plays an important role in RASSF1A regulation. Skp2, a component of the SCF^Skp2^ complex, interacted with RASSF1A and enhanced the ubiquitination of RASSF1A, therefore promoting its degradation [57]. Interestingly, data from our lab also demonstrated that EBNA3C could enhance degradation of two tumor suppressor proteins pRb and p27^Kip1^ by directly interacting with Skp2 and other SCF components [58, 59]. RASSF1A has been implicated in the regulation of the p53–Mdm2 pathway by binding to Mdm2 and Daxx [60]. The association of Mdm2, DAXX, and the deubiquitinase, HAUSP enhances the self-ubiquitin ligase activity of MDM2, which blocks the degradation of p53 in response to DNA damage [60]. Conversely, p53 also inactivated RASSF1A by binding to its promoter and recruiting Daxx and DNMT1 for DNA methylation [61]. Evidence to date has demonstrated that EBNA3C could interact with Mdm2 and p53 simultaneously to form a stable complex [24, 62, 63].

In this study, we explored the mechanism by which EBV regulates RASSF1A expression and its function. We investigated the role of EBNA3C in modulating RASSF1A expression in B-cells, and further explored the methylation status of its promoter. Our study now provides a more comprehensive understanding of the EBNA3C-mediated regulation of cellular functions through its regulation of RASSF1A, and facilitates additional therapeutic avenues to target EBV-associated cancers.

## Results

### EBNA3C expression leads to a reduction in RASSF1A protein levels in human cells

**Fig 1:**
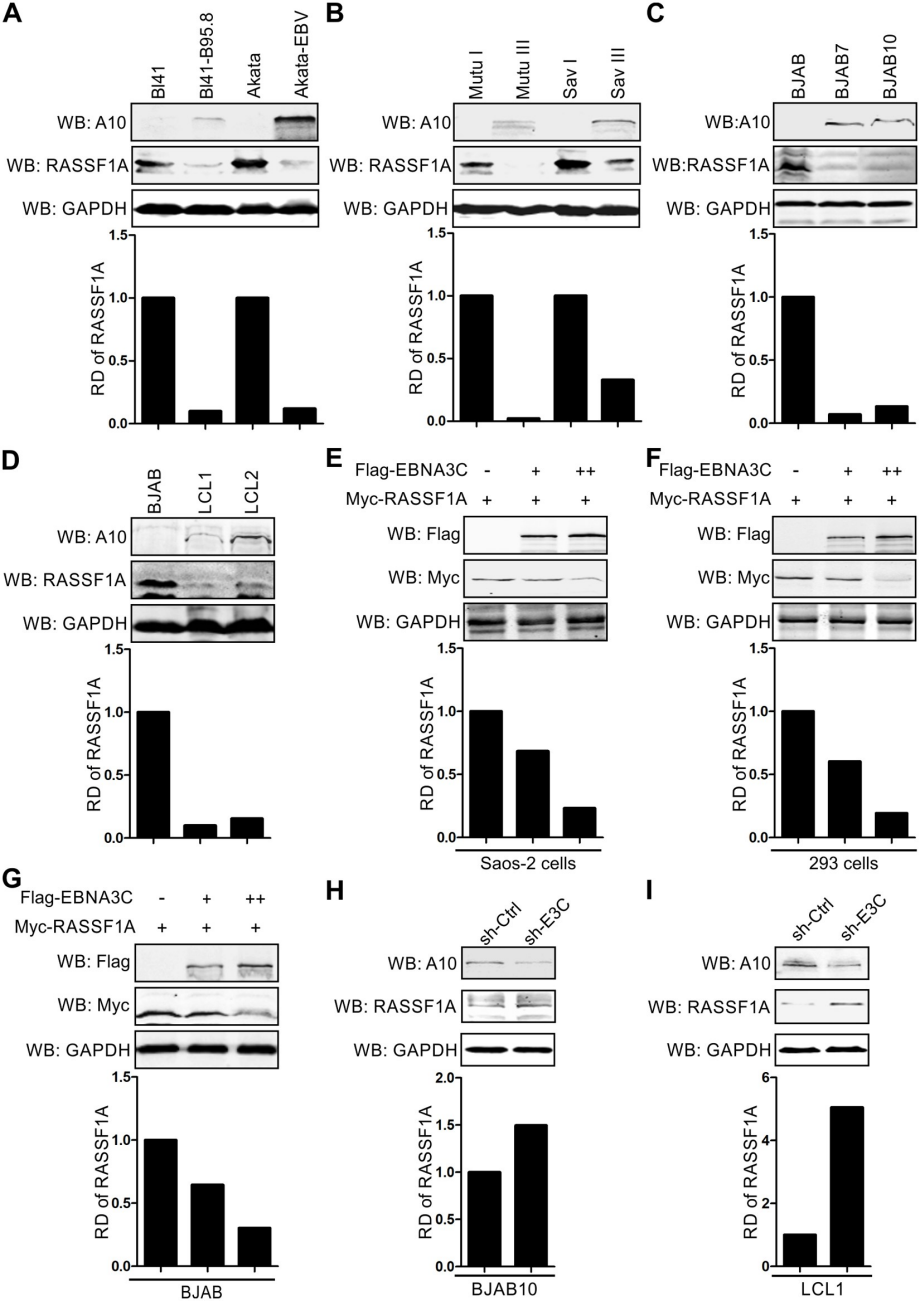
EBNA3C specifically down-regulated RASSF1A protein expression. (A-B) RASSF1A expression was decreased in EBV positive cell lines. 15 million A) Burkitt’s lymphoma cell BL41 and Akata B) Mutu I and Sav I cells and corresponding EBV positive A) BL41-B95.8 and Akata-EBV B) Mutu III and Sav III cells were harvested and lysed with RIPA buffer. The expression levels of RASSF1A were detected by western blot. The relative density (RD) of RASSF1A was quantitated and shown. (C-D) RASSF1A expression was repressed in EBNA3C stably expressing BJAB cells, and EBV-transformed LCLs. The expression of RASSF1A in C) BJAB7 and BJAB10 and D) LCL1 and LCL2 cells were detected as described for A. (E-G) EBNA3C decreased RASSF1A expression specifically. E) Saos-2, F) 293 and G) BJAB cells were transfected with Myc-tagged RASSF1A alone or co-transfected with an increasing amount of Flag-tagged EBNA3C. Western blots were performed to detect the expression of RASSF1A as described for A. (H-I) Knocking down EBNA3C rescued the expression level of RASSF1A in BJAB10 and LCL cells. H) 10 million BJAB10 cells were transfected with sh-EBNA3C or scramble control (sh-Ctrl) by electroporation. I) 15 million lentivirus transduced stable EBNA3C knocked-down LCL1 cells and LCL1-sh-Ctrl were harvested. 48h post-transfection, the cells were harvested and lysed for western blot. The relative density (RD) of RASSF1A was quantitated and shown.

Previous data demonstrated that EBV infection in nascent B-lymphocytes alters methylation status of RASSF1A promoter region [39]. To test whether EBV infection regulates RASSF1A expression, the protein level of RASSF1A was determined in multiple EBV-negative and positive cell lines by western blot assays. We show that RASSF1A protein levels were dramatically decreased in EBV-positive BL41-B95.8, Akata-EBV, Mutu III and Sav III cells when compared with the matching isogenic EBV-negative BL41, Akata, Mutu I and Sav I cells (Fig 1A and 1B). Densitometric analyses showed a drop in greater than 5-fold as seen in the EBV positive cells and type III latency cells Mutu III and Sav III (Fig 1A and 1B). These results suggested that EBV infection inhibits the expression of RASSF1A in these human Burkitt’s cell lines. Additionally, the type I latency cells Mutu I and Sav I did not show a reduction in RASSF1A suggesting that this may be due to a latent antigen expressed in the latency III program.

To examine whether the reduction of RASSF1A expression was mediated by EBNA3C, one of the major viral oncoproteins in latency III program, western blot analysis was performed in EBNA3C expressing stable BJAB7 and BJAB10, and EBV-transformed lymphoblastoid LCL1 and LCL2 cell lines. The results clearly showed that EBNA3C can strongly suppress the expression of RASSF1A by approximately 70% or greater based on the western blot signal (Fig 1C and 1D).

Next, to determine whether RASSF1A expression was specifically regulated by EBNA3C, a dose-dependent increase of EBNA3C was transfected with a constant amount of RASSF1A in three different cell lines—Saos2 (p53^-/-^), HEK293 (p53 is functionally challenged due to adenovirus E1A expression) and BJAB (mutant p53) (Fig 1E–1G). The corresponding western blot data demonstrated that EBNA3C expression caused a dose-dependent decrease in RASSF1A protein levels in all three cell lines, irrespective of p53 expression and function (Fig 1E–1G). To further demonstrate a role for EBNA3C in regulating RASSF1A expression, EBNA3C was knocked-down by expressing a specific EBNA3C short hairpin RNA (sh-E3C) in both EBNA3C stably expressing BJAB cells (BJAB10) and EBV transformed LCL1 (Fig 1G and 1H). Compared to the control lines (sh-Ctrl), RASSF1A expression was significantly rescued in sh-E3C expressing cells (Fig 1G and 1H). Taken together, these results strongly support a role for EBNA3C in suppressing RASSF1A expression.

### EBNA3C associates with RASSF1A in EBV-transformed lymphoblastoid cells

**Fig 2:**
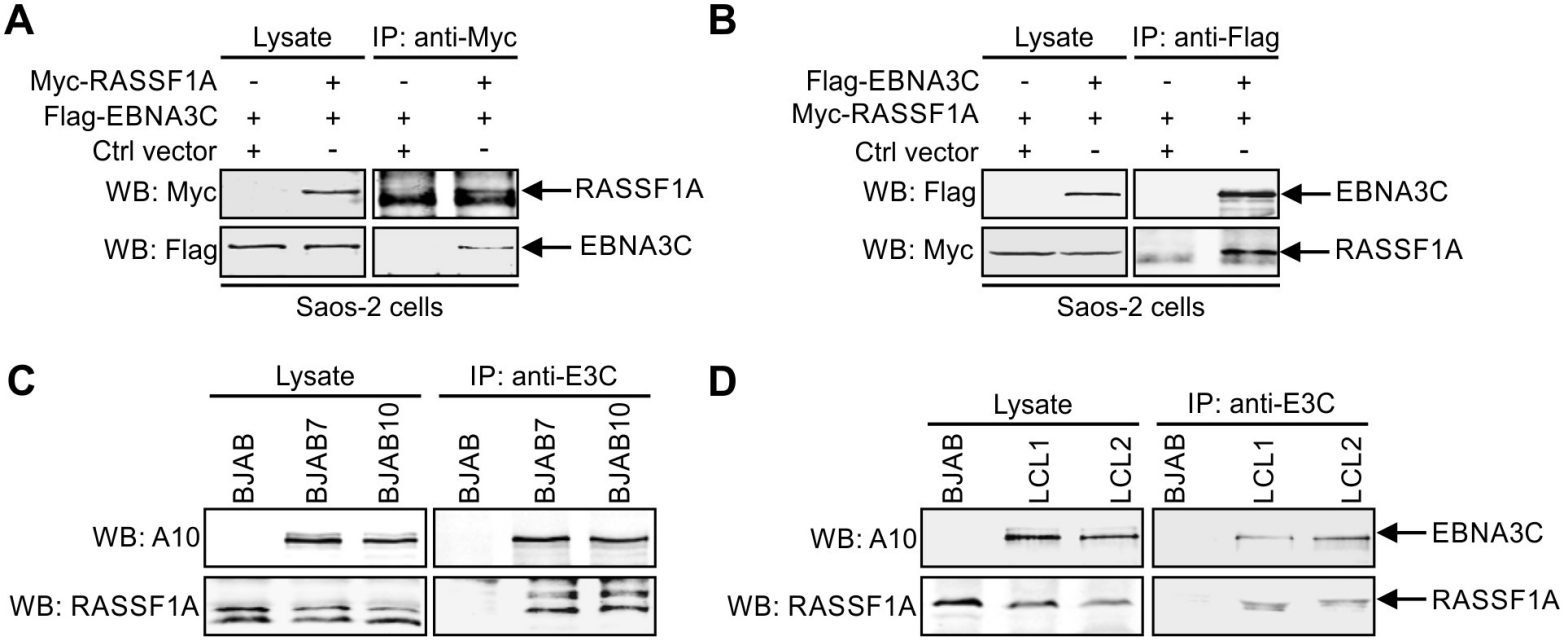
EBNA3C associates with RASSF1A. (A-B) 10 million Saos-2 cells were transfected with Myc-tagged RASSF1A alone or together with Flag-tagged EBNA3C. 48h post-transfection, the cells were harvested and lysed for immunoprecipitation with 1 μg A) anti-Myc or B) anti-Flag antibody. The inputs and immunoprecipitated samples fractionated and specific signals were detected by western blot by using antibodies against Flag and Myc. (C-D) EBNA3C associated with endogenous RASSF1A. 60 million C) BJAB, BJAB7, BJAB10, D) LCL1 and LCL2 were collected and lysed for immunoprecipitation with 1 μg anti-EBNA3C antibody. Western blot was used to detect specific signal in the inputs and immunoprecipitated samples.

EBNA3C mediated suppression of RASSF1A expression prompted us to investigate whether these two proteins can associate with each other as a complex in cells. To this end, co-immunoprecipitation assays were performed in different cell types to determine whether EBNA3C can form a complex with RASSF1A. Flag-tagged EBNA3C and Myc-tagged RASSF1A were co-expressed in Saos-2 cells. The Co-IP assays were carried out by using anti-Myc antibody and Flag antibody, separately. The Co-IP results clearly showed that EBNA3C formed a complex with RASSF1A in cells (Fig 2A and 2B). To further demonstrate the endogenous association between EBNA3C and RASSF1A in the B-cell background, co-immunoprecipitation assays were performed in EBNA3C stably expressed BJAB7 and BJAB10 cells and EBV-transformed LCL1 and LCL2 cells. The results showed that EBNA3C was present in a complex with RASSF1A in the background of B-cells and in EBV-transformed lymphoblastoid cells expressing EBNA3C (Fig 2C and 2D).

### The amino terminus of EBNA3C and carboxy terminus of RASSF1A are important for the association between EBNA3C and RASSF1A

**Fig 3:**
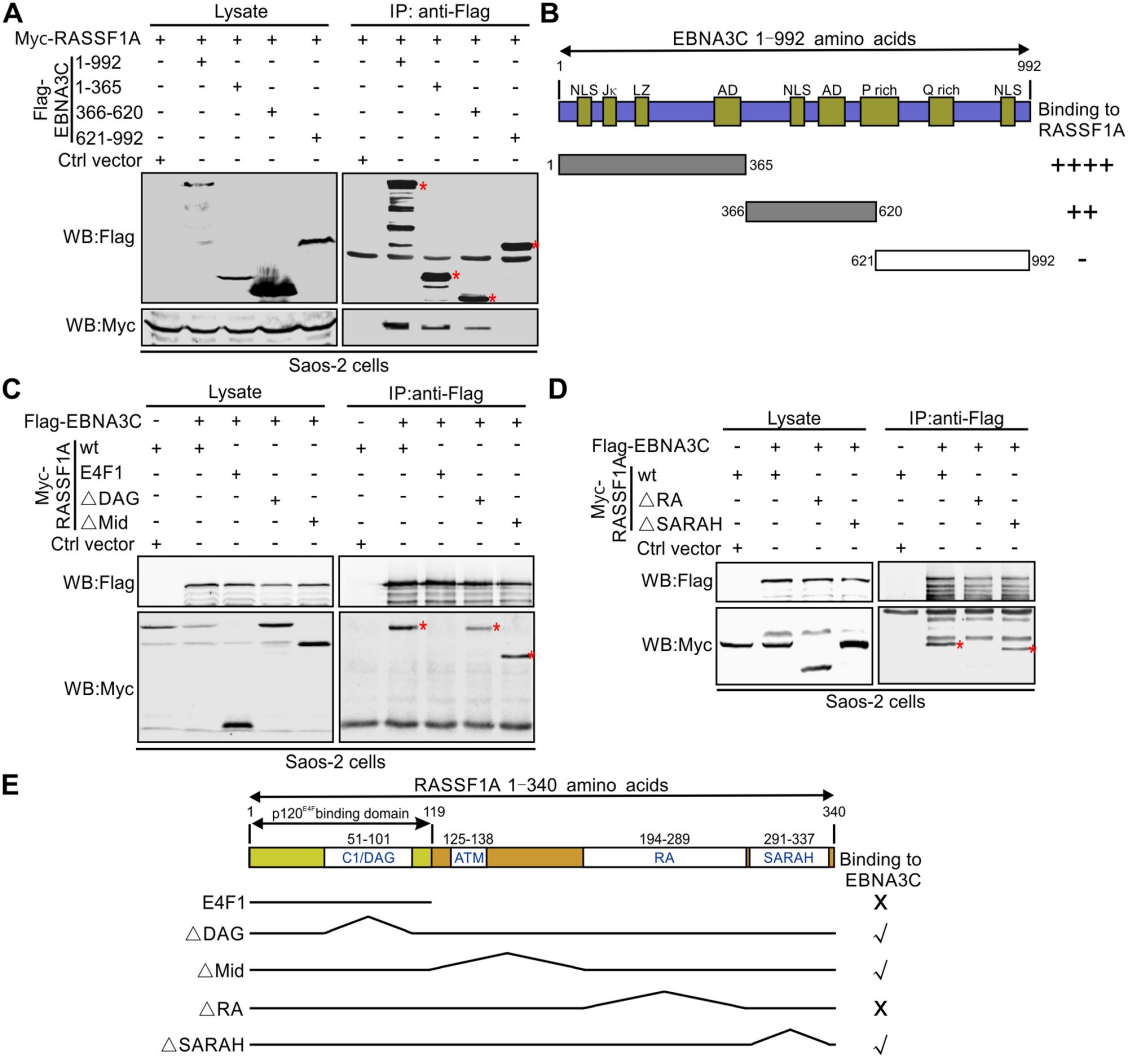
The N-terminus of EBNA3C and C-terminus of RASSF1A are required for the interaction between EBNA3C and RASSF1A. (A) The N terminus of EBNA3C is critical for EBNA3C and RASSF1A interaction. 10 million Saos-2 cells were transfected with Myc-tagged RASSF1A alone or together with Flag-tagged full-length EBNA3C or EBNA3C truncated mutants. 48h post-transfection, the cells were harvested and lysed for immunoprecipitation with 1 μg anti-Flag antibody. The input and immunoprecipitated samples were detected by western blot as described for Fig 2B. (B) The schematic diagram summarizes the binding domains between different regions of EBNA3C and RASSF1A. Jκ, RBP-Jκ; LZ, leucine zipper domain; AD, acidic domains; P rich, Proline-rich; Q rich, glutamine-proline-rich. NLS, nuclear localization signal. -, no binding; ++, binding; ++++, strongly binding. (C-D) The C-terminus of RASSF1A is important for EBNA3C and RASSF1A interaction. 10 million Saos-2 cells were transfected with Myc-tagged RASSF1A truncated mutants and Flag-tagged full-length EBNA3C. 48h post-transfection, the cells were harvested and lysed for immunoprecipitation as described for A. (E) The schematic diagram summarizes the binding domains between different regions of RASSF1A and EBNA3C. E4F1, E1A-regulation transcription factor p120^E4F^; DAG, Conserved region 1 diacylglycerol-binding domain; ATM, ATM-kinase consensus phosphorylation domain; RA, Ras association domain; SARAH, Sav/RASSF/Hpo interaction domain; X, no binding; √, binding.

To map the specific region(s) of EBNA3C important for interaction with RASSF1A, Flag-tagged full length EBNA3C and three domains (1-365aa, 366-620aa and 621-920aa) were expressed in Saos-2 cells. Results from our Co-IP assays indicated that the residues 1–620 of EBNA3C were important for the interaction with RASSF1A (Fig 3A). Although domains which include the activation and other functional motifs showed lesser interaction, the predominant region with greater interaction was residues 1–365. However, the 366–620 residues also showed about 50% of the observed signal compared to the 1–365 amino acid residues (Fig 3A). These results demonstrated that the amino acid residues 1-620aa of EBNA3C were critical for EBNA3C and RASSF1A association (Fig 3B). Next, deleted or truncated RASSF1A mutants were generated and their interaction with full-length EBNA3C were determined. Co-IP experiments were performed by co-expressing Flag-tagged full-length EBNA3C and the different truncated RASSF1A mutants (E4F1, ΔDAG, ΔMid, ΔRA, ΔSARAH) in Saos-2 cells (Fig 3C and 3D). The results demonstrated that the RA region in the C-terminus of RASSF1A was indispensable for its association with EBNA3C (Fig 3E).

### RASSF1A was translocated and co-localized with EBNA3C in nuclear compartments in EBV transformed LCLs

**Fig 4:**
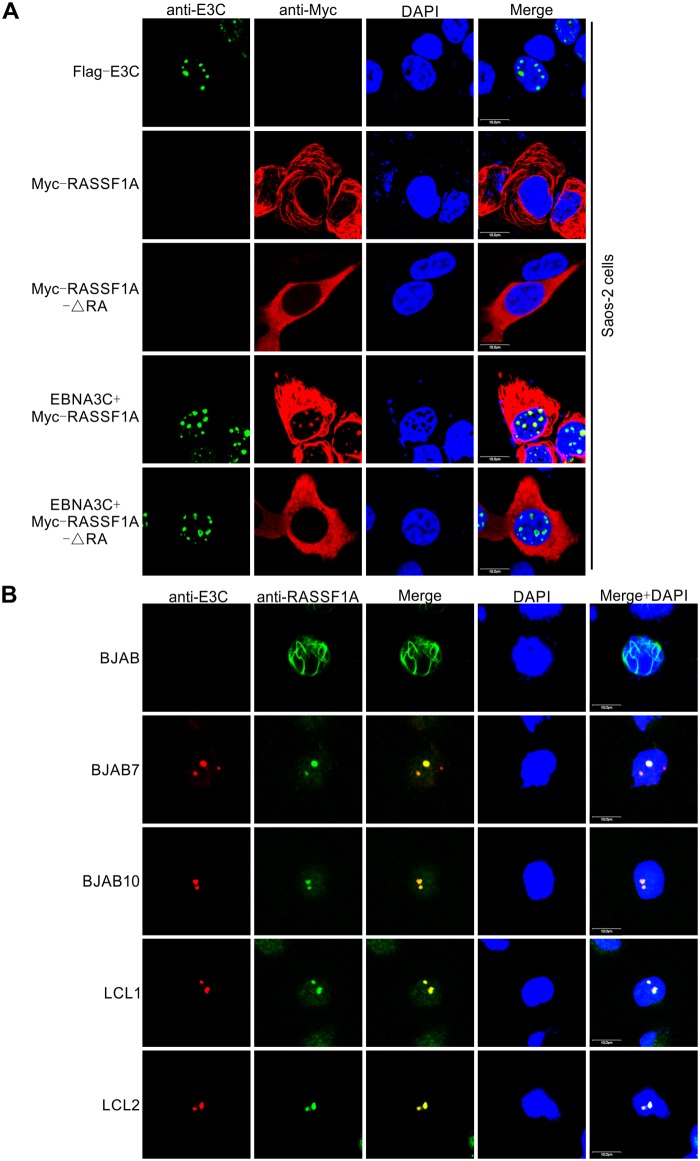
EBNA3C co-localizes with RASSF1A in nuclear compartments in human cells. (A) EBNA3C co-localized with RASSF1A in Saos-2 cells. 0.1 million Saos-2 cells were plated on coverslips and transfected with Flag-tagged EBNA3C, Myc-tagged RASSF1A or RASSF1A-ΔRA separately or together by using jetPRIME transfection reagent. 24h post-transfection, cells were subjected to immunofluorescence assays. (B) EBNA3C co-localized with endogenous RASSF1A in B-cells. BJAB, BJAB7, BJAB10, LCL1, and LCL2 cells were plated on the slide and air-dried. The cells were fixed and subjected to immunofluorescence assays as described in materials and methods.

Since we demonstrated that EBNA3C specifically associated with RASSF1A in cells, immunofluorescence assays were performed to examine the subcellular localization of these two proteins. Flag-tagged EBNA3C was transfected alone or co-transfected with Myc-tagged full-length RASSF1A or RASSF1A-ΔRA in Saos-2 cells (Fig 4A). Specific antibodies against EBNA3C and the Myc-epitope were used in the immunofluorescence analyses. Our results showed that the localization of RASSF1A was predominantly in the cytoplasm when it was transfected alone. However, in the presence of EBNA3C, RASSF1A signals co-localized with EBNA3C in punctate dots in the nucleus (Fig 4A). RASSF1A-ΔRA, which fails to interact with EBNA3C in Co-IP assays was also observed in the cytoplasm and did not co-localize with EBNA3C in the nucleus when co-expressed with EBNA3C. To further support our results, we extended the immunofluorescence assays in the context of B-cells and EBV transformed cells. Specific antibodies against EBNA3C and RASSF1A were used to examine the localization of the endogenous EBNA3C and RASSF1A proteins. Interestingly, EBNA3C specifically co-localized with RASSF1A in the nucleus in two EBNA3C stably expressing BJAB7 and BJAB10 cell lines and two EBV-transformed LCLs (Fig 4B). These results provided additional evidence demonstrating that EBNA3C co-localized with RASSF1A in nuclear compartments as a complex in human cells.

### EBNA3C regulates RASSF1A expression through the ubiquitin-proteasome-dependent pathway

**Fig 5:**
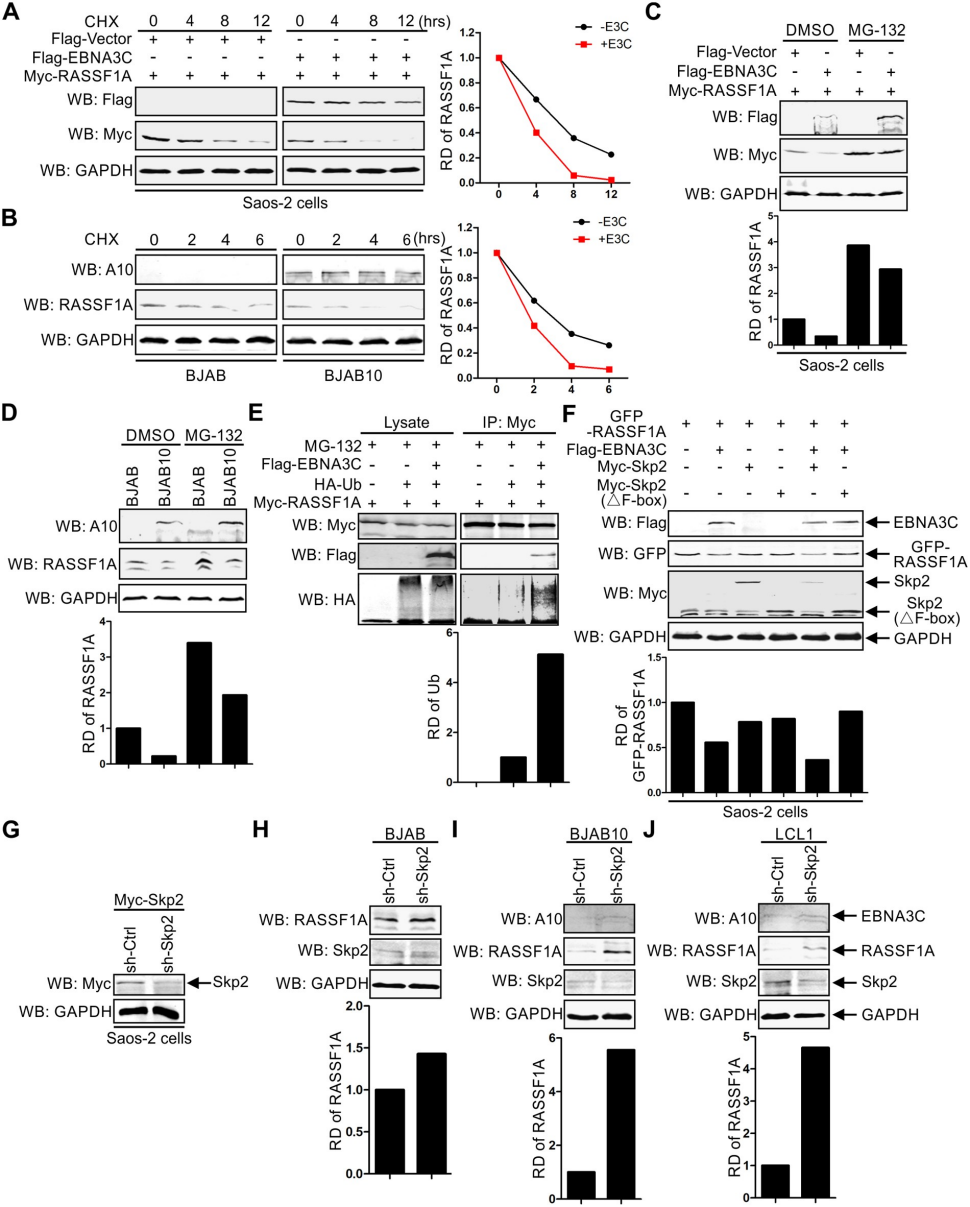
EBNA3C modulates RASSF1A expression in a ubiquitin-proteasome-dependent manner. A-B) EBNA3C decreased RASSF1A stability. (A) 10 million Saos-2 cells were transfected with Myc-tagged RASSF1A and Flag-tagged vector or Flag-tagged EBNA3C. 24 h post-transfection, cells were treated with 40 μg/ml Cycloheximide. (B) 10 million BJAB and BJAB10 cells were treated with 40 μg/ml Cycloheximide. At the indicated time points, the cells were harvested and lysed for western blot. The relative density (RD) of RASSF1A was quantitated to measure RASSF1A rate of degradation. (C-D) EBNA3C decreased RASSF1A stability. C) 10 million Saos-2 cells were transfected with Myc-tagged RASSF1A alone or together with Flag-tagged vector or Flag-tagged EBNA3C. 24 h post-transfection, cells were incubated with 20 μM MG132 for another 16 hours. D) 10 million BJAB and BJAB10 cells were incubated with 20 μM MG132 for 16 hours. Then cells were harvested and RASSF1A expression was detected by western blot. (E) EBNA3C enhanced RASSF1A poly-ubiquitination. 10 million Saos-2 cells were transfected with the indicated constructs. 24 h post-transfection, cells were incubated with 20 μM MG132 for another 16 hours. Then the cells were harvested and subjected to immunoprecipitation using an antibody against Myc. The inputs and immunoprecipitated samples were detected by western blot. (F) Skp2 protein was recruited by EBNA3C to mediate RASSF1A degradation. GFP-tagged RASSF1A was co-transfected with plasmids encoding EBNA3C and/or Skp2 or Skp2 truncated mutant. 24 h post-transfection, cells were harvested and lysed for western blot. The expression of RASSF1A, EBNA3C, and Skp2 was detected with specific antibodies. (G) The effect of sh-Skp2 in reducing Skp2 expression was detected in Saos-2 cells. Myc-tagged Skp2 and sh-Ctrl, or sh-Skp2 were transfected into Saos-2 cells. 48 hours post-transfection, the cells were harvested and the expression of Skp2 was detected by western blot. (H-J) Silencing Skp2 rescued RASSF1A protein expression. Skp2 knocked down BJAB H), BJAB10 I), and LCL1 cells J) were constructed by lentiviruses and selected by puromycin. 3 days later, the cells were harvested and lysed for western blot analyses. The expression levels of RASSF1A, EBNA3C, Skp2, and GAPDH were detected. The relative density (RD) of RASSF1A was quantitated and shown.

EBNA3C is known to modulate the ubiquitin (Ub)-proteasome machinery and regulates the stabilization of many oncoproteins, cellular kinases, and transcription factors [24, 64, 65]. To test whether EBNA3C-mediated RASSF1A down-regulation was due to modulation of the ubiquitin-proteasome machinery, a stability assay was performed by expressing RASSF1A with or without EBNA3C in the presence of the protein synthesis inhibitor cycloheximide (CHX). Saos-2 cells were transfected with Myc-tagged RASSF1A and Flag-tagged EBNA3C or Flag-tagged empty vector. 24 hours post-transfection, the transfected cells were treated with CHX and the expression of RASSF1A was detected by western blot at 0, 4, 8, 12 hours. The results demonstrated that RASSF1A expression levels were markedly reduced in the presence of EBNA3C. Whereas, RASSF1A was more stable in the absence of EBNA3C (Fig 5A). To further validate this observation, we extended the stability assays to EBNA3C negative BJAB and EBNA3C stably expressing BJAB10 cells. BJAB and BJAB10 cells were incubated with CHX for 0, 2, 4, 6 hours. The endogenous RASSF1A was monitored by western blot assays. As expected, our results showed that RASSF1A protein levels were significantly reduced in BJAB10 cells by 6 hours with the treatment of cycloheximide (Fig 5B).

Previous studies showed that RASSF1A protein was degraded via the ubiquitin-mediated proteasome [57]. Therefore, it was expected that the increased instability of RASSF1A was likely due to the effects of EBNA3C-related ubiquitin-proteasome machinery as EBNA3C is able to recruit E3 ligases to degrade targeted cellular substrates [66]. To test this possibility, Myc-tagged RASSF1A was transfected with Flag-tagged EBNA3C or empty vector in Saos-2 cells. 24 hours post-transfection, the cells were incubated with the proteasome inhibitor MG132 or DMSO for 16 hours. The results showed a substantial accumulation of RASSF1A protein in MG132 treated cells, even in the presence of EBNA3C, compared with that seen in mock treated and control vector groups (Fig 5C). To further corroborate our results, we treated BJAB and BJAB10 cells with MG132 for 16 hours. Similar results were observed in MG132 treated B cells (Fig 5D). Together, these results suggest that the instability of RASSF1A was regulated by EBNA3C in association with the ubiquitin-proteasome pathway.

Next, we performed in vivo poly-ubiquitination assays to further investigate the role of EBNA3C in regulating RASSF1A poly-ubiquitination. Saos-2 cells were co-transfected with Myc-tagged RASSF1A, HA-tagged Ubiquitin, with or without Flag-tagged EBNA3C. 24 hours later, the cells were treated with MG132 for another 16 hours. Specific antibody against the Myc-epitope was used in the following immunoprecipitation analysis. Enhanced poly-ubiquitination levels of RASSF1A were observed in the presence of EBNA3C when compared with that in RASSF1A and control vector or the HA-tagged Ubiquitin co-expressed group (Fig 5E). This result further corroborated the above results and strongly suggested that RASSF1A was degraded via the ubiquitin-proteasome pathway mediated by EBNA3C.

### EBNA3C recruits Skp2 to enhance RASSF1A degradation

Earlier studies have shown that Skp2, a critical E3 ubiquitin ligase, was associated with RASSF1A and promoted RASSF1A ubiquitination and degradation [57]. Moreover, previous studies showed that Skp2 directly interacted with EBNA3C and facilitated the degradation of p27, E2F, c-Myc and the retinoblastoma protein [58, 59, 67, 68]. These studies prompted us to test the possible role of Skp2 in EBNA3C-enhanced RASSF1A degradation. Wild-type Skp2 and the functional deficient mutant, Skp2 lacking the so-called F box domain, were co-expressed with EBNA3C and RASSF1A, separately. The following western blot results showed that when RASSF1A were co-expressed with EBNA3C, the protein levels were substantially reduced close to about 60% compared with RASSF1A expressed alone (Fig 5F). Skp2 and Skp2(ΔF-box) also decreased the expression of RASSF1A, but less dramatically. These results indicated that Skp2 has a limited role in regulating RASSF1A expression when Skp2 was expressed with RASSF1A alone. However, when RASSF1A was co-expressed with EBNA3C and Skp2, the protein levels of RASSF1A decreased sharply close to approximately 35%, while the RASSF1A protein levels were not changed and even slightly increased when it was co-expressed with EBNA3C and Skp2(ΔF-box) (Fig 5F). The protein levels of Skp2 also decreased when it was co-expressed with RASSF1A and EBNA3C, but no obvious change in protein levels was observed for the Skp2 (ΔF-box) (Fig 5F). To further examine the role of Skp2 in modulating RASSF1A protein expression, an sh-RNA targeted Skp2 was constructed and the effect of sh-Skp2 was tested in Saos-2 cells by co-transfecting Myc-tagged Skp2 with sh-Skp2. The expression of Myc-tagged Skp2 was dramatically decreased in sh-Skp2 transfected cells (Fig 5G). Then the effect of Skp2 on RASSF1A protein expression was determined in BJAB, BJAB10, and LCL1 cell lines. The results showed that silencing Skp2 in BJAB cells led to an increase in RASSF1A expression (Fig 5H). Furthermore, in BJAB10 and LCL1cells, knocking down Skp2 significantly increased RASSF1A expression (Fig 5I and 5J). This suggested that Skp2 played an important role in EBNA3C-associated RASSF1A downregulation. These results demonstrated that EBNA3C can enhance Skp2 mediated RASSF1A degradation through the ubiquitin-proteasome degradation pathway.

### EBNA3C suppresses RASSF1A mRNA expression by enhancing methylation of its promoter

**Fig 6:**
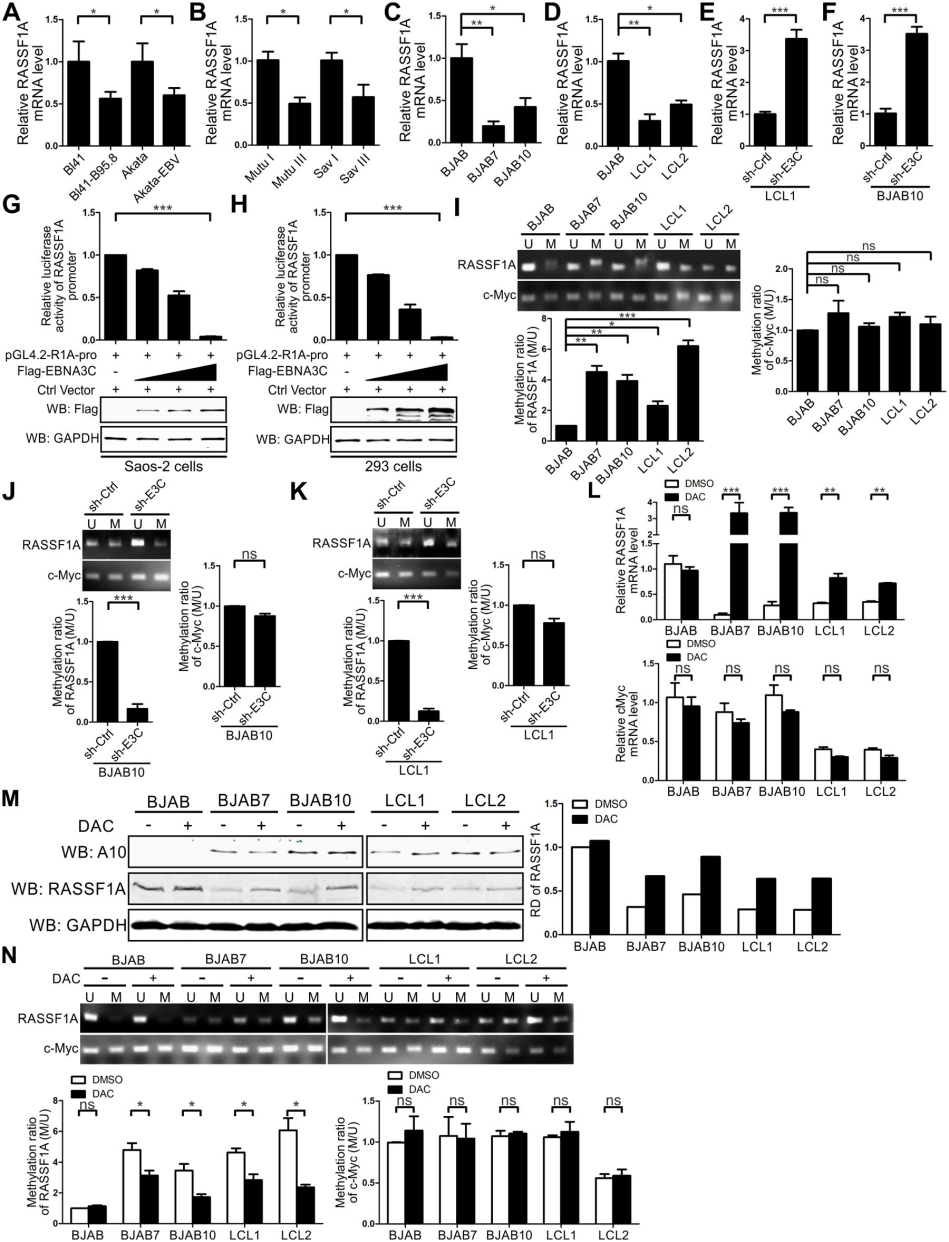
EBNA3C down-regulated RASSF1A mRNA expression by enhancing RASSF1A promoter methylation. (A-D) RASSF1A mRNA expression was decreased in A-B) EBV positive cell lines and C-D) BJAB7, BJAB10, LCL1, and LCL2 cells. 5 million B-cells were collected and total RNA extracted via Trizol reagent. cDNAs were generated by reverse transcriptase kit and RASSF1A mRNA expression level was detected by real-time PCR. The mRNA expression of GAPDH was set as a control. Each sample was determined in triplicate. (E-F) Knock-down EBNA3C rescued RASSF1A mRNA expression. The mRNA expression of RASSF1A in EBNA3C knock-downed LCL1 cell lines or in sh-EBNA3C transfected BJAB10 cells was determined as described for A-D. (G-H) EBNA3C decreased the transcriptional activity of the RASSF1A promoter. 0.5 million Saos-2 and 293 cells were transfected with pGL4.2-R1A-promoter, pRL-TK and an increasing amount of Flag-tagged EBNA3C. Total amounts of plasmids were kept constant by co-transfecting with the vector. 36 h post-transfection, cells were harvested and lysed for dual-luciferase reporter assay according to the manufacturer’s instructions and the expression of Flag-tagged EBNA3C was detected by western blot. All assays were repeated at least three times for reproducibility. (I) The methylation levels of the RASSF1A promoter was specifically increased in BJAB7, BJAB10, LCL1, and LCL2 cells. 5 million BJAB, BJAB7, BJAB10, LCL1, and LCL2 cells were harvested and genomic DNA was extracted as described in materials and methods. 1 μg genomic DNA was used for bisulfite conversion and purification via the EZ DNA Methylation Gold Kit. 100 ng and 50 ng bisulfite-modified DNA was used in 15 μl methylation-specific PCR (MSP) reaction to amplify methylated (M) and unmethylated (U) DNA fragments. The relative density of methylated (M) and unmethylated (U) DNA fragments was measured using Image J software. The ratio of methylated (M) DNA fragments to unmethylated (U) DNA fragments of RASSF1A or c-Myc promoter in BJAB cells was set as the basic level separately. The methylation ratio was calculated by comparing the ratio of methylated (M) DNA fragments to unmethylated (U) DNA fragments of RASSF1A or c-Myc promoter in BJAB7, BJAB10, LCL1 and LCL2 cells to that in BJAB cells. Mean values and standard deviation of two independent experiment were presented. (J-K) Knockdown of EBNA3C decreased the methylation level of RASSF1A promoter. The methylation ratio of the RASSF1A and c-Myc promoter in LCL1-sh-EBN3C and sh-EBNA3C transfected BJAB10 cells were determined as described above. (L-N) RASSF1A mRNA, protein expression levels and methylation status were specifically increased in DAC-treated B cells. 5 million BJAB, BJAB7, BJAB10, LCL1, and LCL2 cells were treated with 5 μM DAC and the medium were refreshed every day. 5 days later, the cells were harvested and aliquoted into three fractions. The mRNA L) and protein M) expression levels of RASSF1A in DAC-treated B cells were detected by real-time PCR and western blot. The mRNA expression level of c-Myc and protein expression level of EBNA3C were determined simultaneously. N) The methylation status of RASSF1A and c-Myc were detected by methylation specific PCR.

Numerous studies have shown that a large number of tumor suppressor genes have been epigenetically modified in EBV infected cells, which resulted in a reduction in their mRNA expression [37]. To further determine whether EBNA3C regulates RASSF1A mRNA expression, endogenous RASSF1A mRNA expression was monitored by real-time PCR in EBV negative and positive cells. The results showed that RASSF1A mRNA expression was down-regulated approximately 2-fold in EBV positive cell lines when compared with EBV negative cell lines as well as type I and III latency cells (Fig 6A and 6B). We further extended the real-time PCR assay in BJAB, BJAB7, BJAB10, LCL1, and LCL2. As anticipated, mRNA expression of RASSF1A was decreased significantly in EBNA3C stably expressing BJAB7, BJAB10 cells, and EBV-transformed LCL1 and LCL2 when compared with that in BJAB cells (Fig 6C and 6D). To further exploit the role of EBNA3C in repressing RASSF1A mRNA expression, real-time PCR assays were performed in EBNA3C stably knocked down LCL1 cells and sh-EBNA3C transfected BJAB 10 cells. The results showed that RASSF1A mRNA expression was dramatically upregulated in EBNA3C knocked-down cells (Fig 6E and 6F). These results demonstrated that EBNA3C can suppress RASSF1A mRNA expression.

To further define how EBNA3C represses RASSF1A mRNA expression, a dual-luciferase assay was performed to detect the transcriptional activity at the RASSF1A promoter in the presence of EBNA3C. The reporter, pGL4.2-RASSF1A-promoter (-629 to 1) was generated and tested. The RASSF1A-promoter reporter and thymidine kinase promoter-Renilla luciferase reporter were transfected into Saos-2 and 293 cells with a dose-dependent increase in Flag-tagged EBNA3C. The results of the luciferase assay suggested that EBNA3C inhibited RASSF1A promoter transcriptional activity in a dose-dependent manner (Fig 6G and 6H).

In a variety of human cancers, RASSF1A was inactivated by epigenetic silencing [39, 40]. In the context of cancer progression, hypermethylation of CpG islands is one of the most common epigenetic modifications, which occurs at the RASSF1A promoter and results in the loss of RASSF1A transcripts [39]. To further explore the mechanism by which EBNA3C inhibited RASSF1A promoter activity, methylation specific PCR assays were performed to detect the methylation status of the RASSF1A promoter by using two different pairs of primers. The c-Myc gene, whose promoter was reported to be hypomethylated [37, 69, 70] was used as a negative control and the methylation status of its promoter was detected simultaneously. The results showed that the methylated ratio of the RASSF1A promoter was increased not only in EBNA3C stably expressing BJAB7 and BJAB10 cells but also in EBV transformed LCL1 and LCL2 cells (Fig 6I). No significant change was detected in the c-Myc promoter (Fig 6I). EBNA3C was reported to interact with c-Myc [71], but it did not increase its methylation levels. These results suggested EBNA3C selectively induced methylation of the RASSF1A promoter. To provide additional evidence that the enhanced methylation status was related to the presence of EBNA3C, RASSF1A promoter methylation levels were determined in EBNA3C stably knocked-down LCL1 cells and sh-EBNA3C transfected BJAB 10 cells. The methylated ratio of the RASSF1A promoter was decreased in EBNA3C knocked down cells compared with the cells transfected with sh-control. However, no obvious change was observed in the c-Myc promoter (Fig 6J and 6K).

5-Aza-2′-deoxycytidine (DAC), a DNA methylation inhibitor reported to lead to demethylation of the 5’CpG island of RASSF1A and re-expression of its transcripts [72–74] was used in our experiments to test whether DAC could relieve the repression of EBNA3C and rescue RASSF1A expression in EBNA3C and EBV positive cell lines. 5 million BJAB, BJAB7, BJAB, LCL1, and LCL2 cells were treated with 5 μM DAC for 5 days. The mRNA and protein expression levels of RASSF1A, and the methylation status of the RASSF1A and c-Myc promoters was examined. The mRNA and protein expression levels of RASSF1A were increased dramatically in DAC-treated EBNA3C and EBV positive cells (Fig 6L and 6M). However, no significant change was observed in c-Myc mRNA expression levels in any of the 5 cell lines (Fig 6L). Methylation specific PCR was performed in DAC-treated B cells to determine the methylation status of the RASSF1A and c-Myc promoter. As expected, the methylation status of the RASSF1A promoter was increased, but there was no obvious change in the c-Myc promoter (Fig 6N). These results further demonstrated that EBNA3C specifically down-regulated RASSF1A mRNA expression by enhancing methylation of its promoter.

### EBNA3C enhances RASSF1A promoter methylation by modulating expression of DNMTs

**Fig 7:**
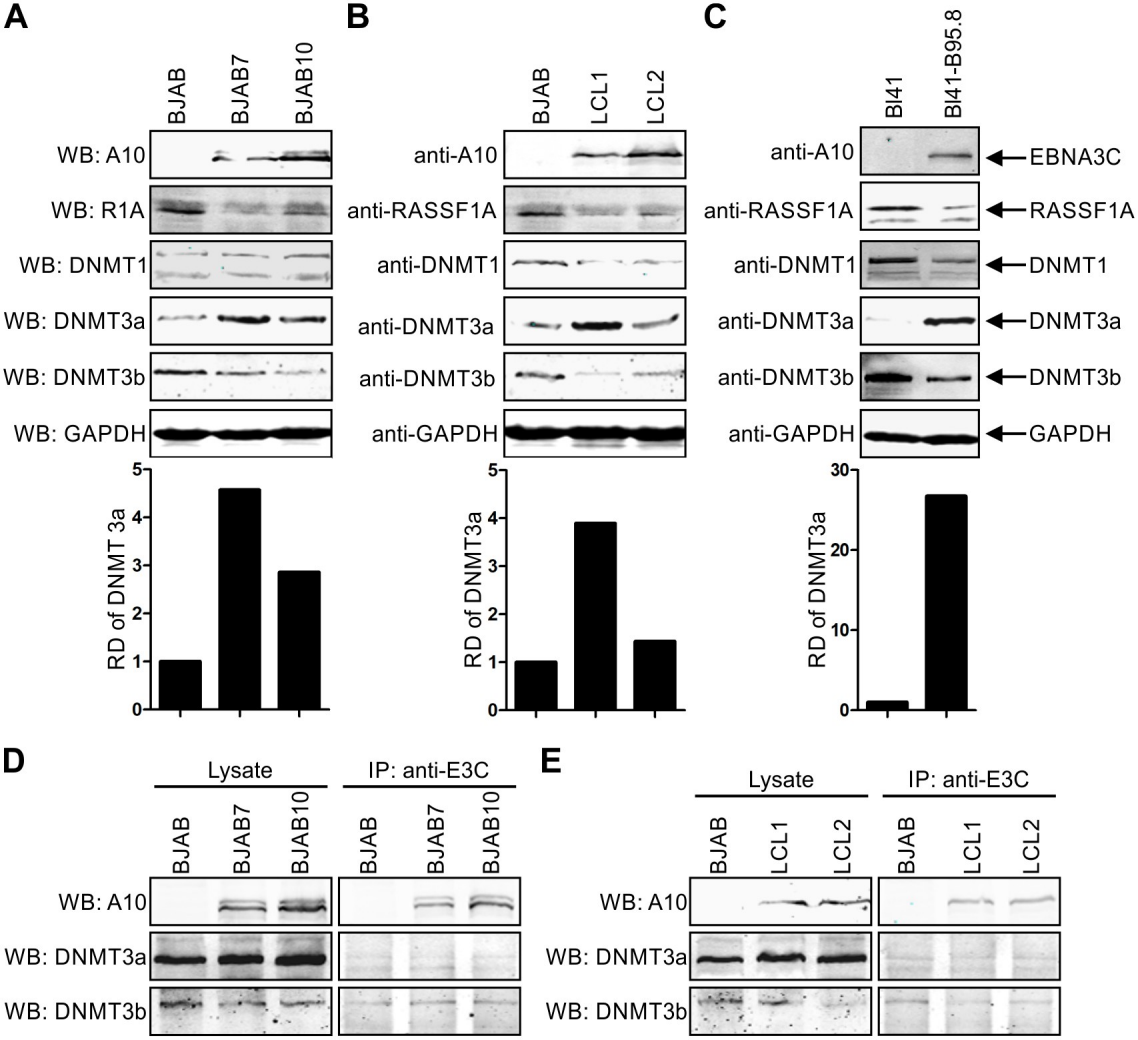
EBNA3C modulated DNMT levels. (A-B) The expression of DNMTs were detected in B cells. 15 million A) BJAB, BJAB7, BJAB10, B) LCL1, LCL2, C) BL41, and BL41-B95.8 were collected and lysed with RIPA buffer. The expression of DNMT1, DNMT3a, and DNMT3b was monitored by western blot with specific antibodies. The relative density (RD) of DNMT3a was quantitated and presented. (D-E) The interaction between EBNA3C and endogenous DNMT3a and DNMT3b were detected in B cells. 60 million D) BJAB, BJAB7, BJAB10, E) LCL1 and LCL2 were collected and lysed for immunoprecipitation with 1 μg anti-EBNA3C antibody. Western blot was used to detect specific signal in the input and immunoprecipitated samples.

Previous data from our lab have shown that EBNA3C could interact with DNMT1 and induce DNMT1 expression [56]. Earlier studies also showed that silencing DNMT1 could upregulate RASSF1A expression and suppress RASSF1A methylation in esophageal squamous cell carcinoma [75]. Further, DNMT1 and other DNMTs are involved in maintaining the overall methylation density in cells [76]. We detected the expression of endogenous DNMT1, DNMT3a and DNMT3b in EBNA3C stably expressing BJAB7 and BJAB10 cells and EBV- transformed LCL1 and LCL2 cells. Western blot results showed that EBNA3C can also increase DNMT1 expression in EBNA3C stably expression BJAB7 and BJAB10 cells (Fig 7A). However, in the context of EBV positive cell lines, DNMT1 and DNMT3b expression levels were decreased in LCL1, LCL2, and BL41-B95.8 (Fig 7A, 7B and 7C). Further, the expression of DNMT3a was most dramatically increased in EBNA3C and EBV positive cells (Fig 7A, 7B and 7C), and was consistent with a previously published report [77]. Importantly, we attempted to detect the interaction between EBNA3C and DNMT3a or DNMT3b, but our results showed little or no obvious interaction (Fig 7D and 7E). Therefore, our results suggested that EBNA3C could up-regulate RASSF1A promoter methylation by modulating expression of DNMTs expression.

### EBNA3C inhibits RASSF1A-mediated cell apoptosis and promotes cell proliferation

**Fig 8:**
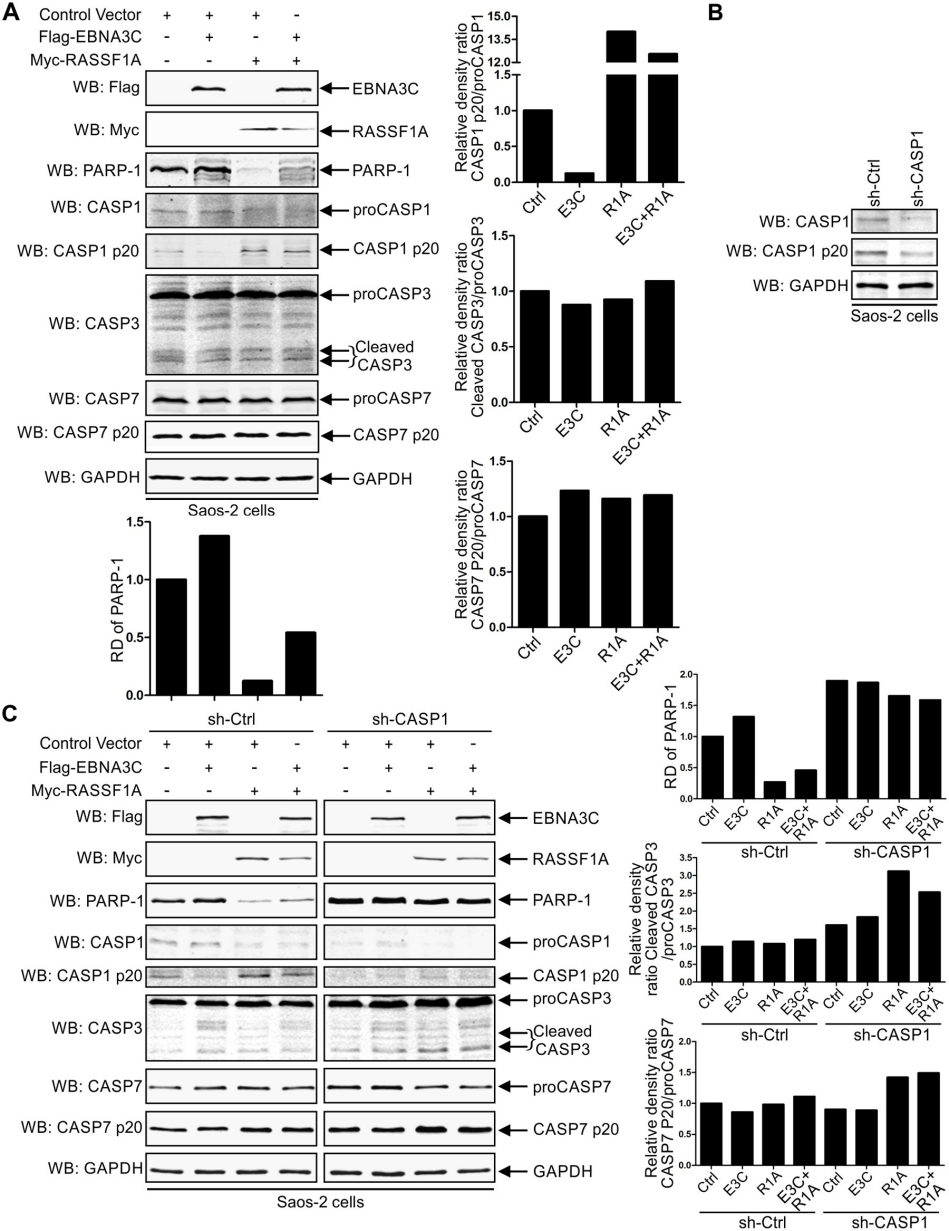
EBNA3C inhibits RASSF1A-mediated cell apoptosis. (A) 10 million Saos-2 cells were transfected with control vector, plasmids encoding EBNA3C, RASSF1A. 48 h post-transfection, cells were harvested and the expression of PARP-1, full-length and cleaved caspase-1, caspase 3 and caspase 7 were detected by western blot. The relative density (RD) of PARP-1 and cleaved caspase products to full-length caspase proteins were quantitated and presented. (B) Short hairpin RNA targeted caspase-1 (sh-CASP1) decreased caspase-1 expression in Saos-2 cells. sh-CASP1 was constructed and transfected into Saos-2 cells. 48 hours post-transfection, the cells were harvested and the expression levels of full-length caspase-1 and cleaved caspase-1 p20 were detected by western blot. (C) The expression of PARP-1 and the cleavage of caspase 3 and caspase 7 were increased in caspase-1 knocked-down Saos-2 cells. Flag-tagged EBNA3C and/or Myc-tagged RASSF1A were transfected with sh-control or sh-caspase-1 separately in Saos-2 cells. 48 hours post-transfection, the cells were harvested. The expression levels of PARP1, the cleavage of caspase-1, caspase-3, and caspase-7 were detected.

RASSF1A was shown to play a role in cell apoptosis by interacting with a variety of cellular proteins [40, 48, 49]. To evaluate the effect of EBNA3C on RASSF1A-mediated cell apoptosis, Saos-2 cells were transfected with Flag-tagged EBNA3C and Myc-tagged RASSF1A. The expression of the apoptotic pathway related proteins was determined by western blot. First, the expression of PARP-1(Poly [ADP-ribose] polymerase 1), involved in DNA damage repair [78, 79] was analyzed. The results showed that cleavage of PARP-1 was significantly higher in RASSF1A expressed cells compared to that in mock transfected cells (Fig 8A). EBNA3C may protect PARP-1 from cleavage, and rescued the expression of PARP-1 when co-expressed with RASSF1A (Fig 8A). It was reported that inactivation of PARP-1 was cleaved by activated caspase-1, caspase 3 or caspase 7. Full-length caspase proteins and their cleaved products were also monitored by western blot. Band intensities were quantified and relative density ratios of the cleaved products to total caspase proteins were determined. The cleavages of caspase 3 and caspase 7 did not show a significant change in RASSF1A expressed cells compared to other groups. However, activated caspase-1 was increased dramatically in RASSF1A expressing cells (Fig 8A). These results suggested that EBNA3C can inhibit RASSF1A-mediated cell apoptosis by utilizing a caspase-1-dependent apoptotic pathway.

To further define the effect of caspase-1 on RASSF1A-mediated cell apoptosis, a short hairpin RNA targeted caspase-1 (sh-CASP1) was constructed. sh-CASP1 effectively decreased caspase-1 and caspase-1 cleaved product expression in Saos-2 cells (Fig 8B). The effects of EBNA3C and RASSF1A on PARP-1 expression and cleavages of caspase proteins were monitored in caspase-1 knocked-down Saos-2 cells. Silencing caspase-1 significantly increased PARP-1 expression. However, EBNA3C failed to significantly increase PARP-1 expression when compared with control in caspase-1 knocked-down Saos-2 cells (Fig 8C). RASSF1A slightly decreased PARP-1 expression when compared with control (Fig 8C). To our surprise, the cleavage of caspase-3 and caspase-7 were increased in RASSF1A expressed cells when caspase-1 was knocked down (Fig 8C). These results suggested that caspase-1 played an important role in RASSF1A-mediated cell apoptosis. However, when caspase-1 was knocked down, RASSF1A may induce the cleavage of caspase-3 and caspase-7 to regulate cell apoptosis.

**Fig 9:**
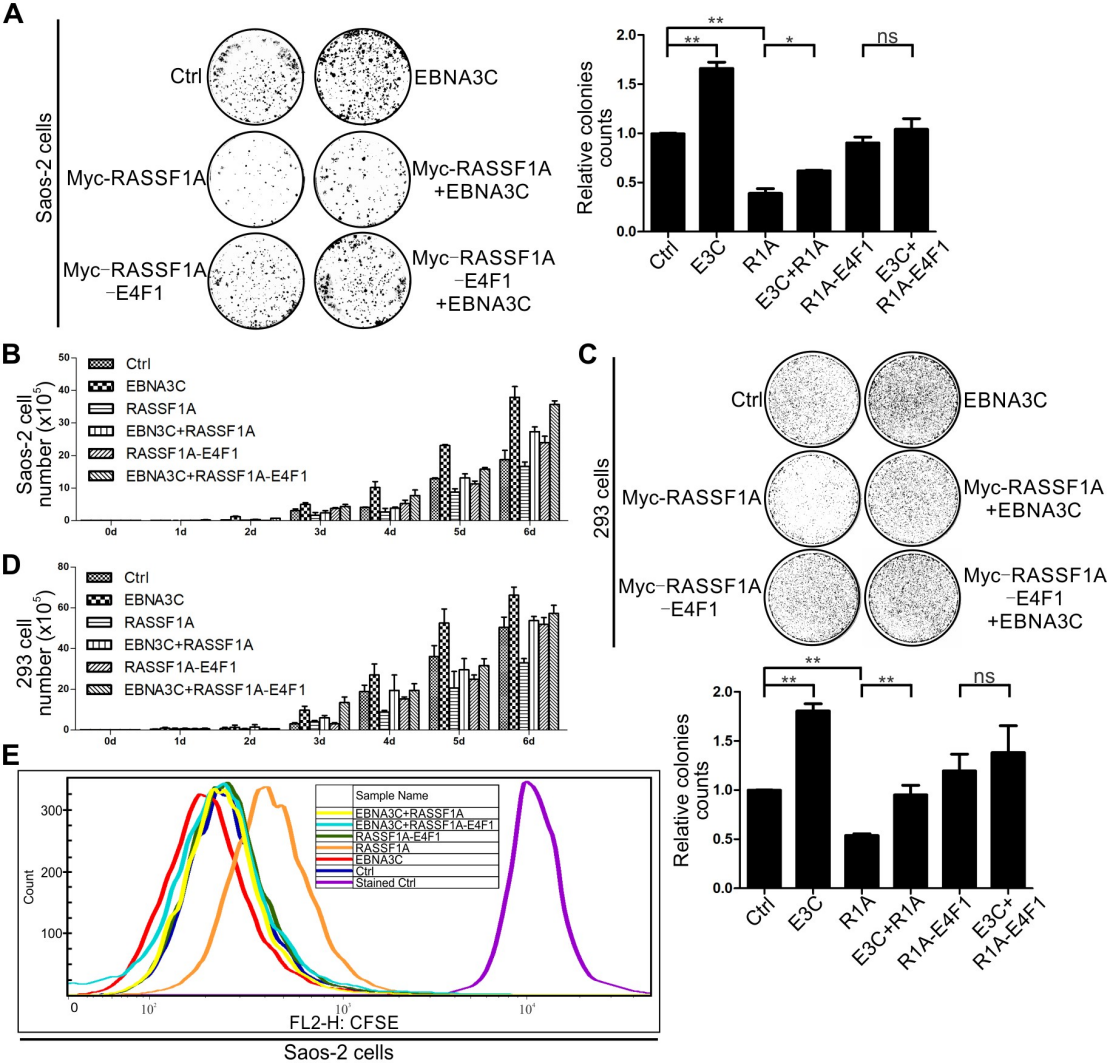
EBNA3C promotes cell proliferation by inhibiting the effect of RASSF1A. (A) Saos-2 cells were transfected with the indicated plasmids and eGFP. The cells were selected with a G418 antibiotic for two weeks. The cells were fixed and the cell colonies were stained with 0.1% crystal violet. The relative colony number was measured by Image J software. (B) 5x10^4^ selected cells were plated and cultured for 6 days. Viable cells were counted every day using trypan blue staining. (C-D) 293 cells were transfected, selected and the viable cells were counted as described above. (E) EBNA3C inhibited RASSF1A activities and promoted cell proliferation. 293 cells were transfected with RASSF1A, RASSF1A-E4F1 singly or co-transfected with EBNA3C. The cells were selected with a G418 antibiotic for two weeks. 1x10^5^ cells were incubated with CFSE and cultured in 37 °C incubator for 3 days. Then the cells were harvested and analyzed by flow cytometry assay.

Next, we examined cell proliferation in EBNA3C and RASSF1A expressing cells. Saos-2 and 293 cells were transfected along with the control vector, RASSF1A, RASSF1A-E4F1 alone or together with EBNA3C and selected with G418 for colony formation. The results showed that a sharp decrease in colony number was seen in RASSF1A transfected Saos-2 cells when compared with mock transfected cells (Fig 9A). EBNA3C rescued the reduction in colony formation when it was co-expressed with RASSF1A. However, no obvious difference was observed between mock transfected cells and RASSF1A-E4F1 transfected cells. EBNA3C moderately increased the colony formation when co-expressed with RASSF1A-E4F1 (Fig 9A). To further corroborate our results, cell proliferation assays were performed in Saos-2 cells and the cell numbers were counted. Consistent with our colony formation results, EBNA3C rescued RASSF1A-mediated inhibition of cell proliferation (Fig 9B). We also repeated these experiments in 293 cells and similar results were obtained (Fig 9C and 9D). The following CFSE assays were performed to detect cell proliferation in RASSF1A, RASSF1A-E4F1, and EBNA3C transfected Saos-2 cells. The results demonstrated that RASSF1A but not the RASSF1A-E4F1 mutant inhibited cell proliferation and that EBNA3C could promote cell proliferation by inhibiting RASSF1A activities (Fig 9E).

### Knockdown of RASSF1A through shRNA induces transformation of LCLs

**Fig 10:**
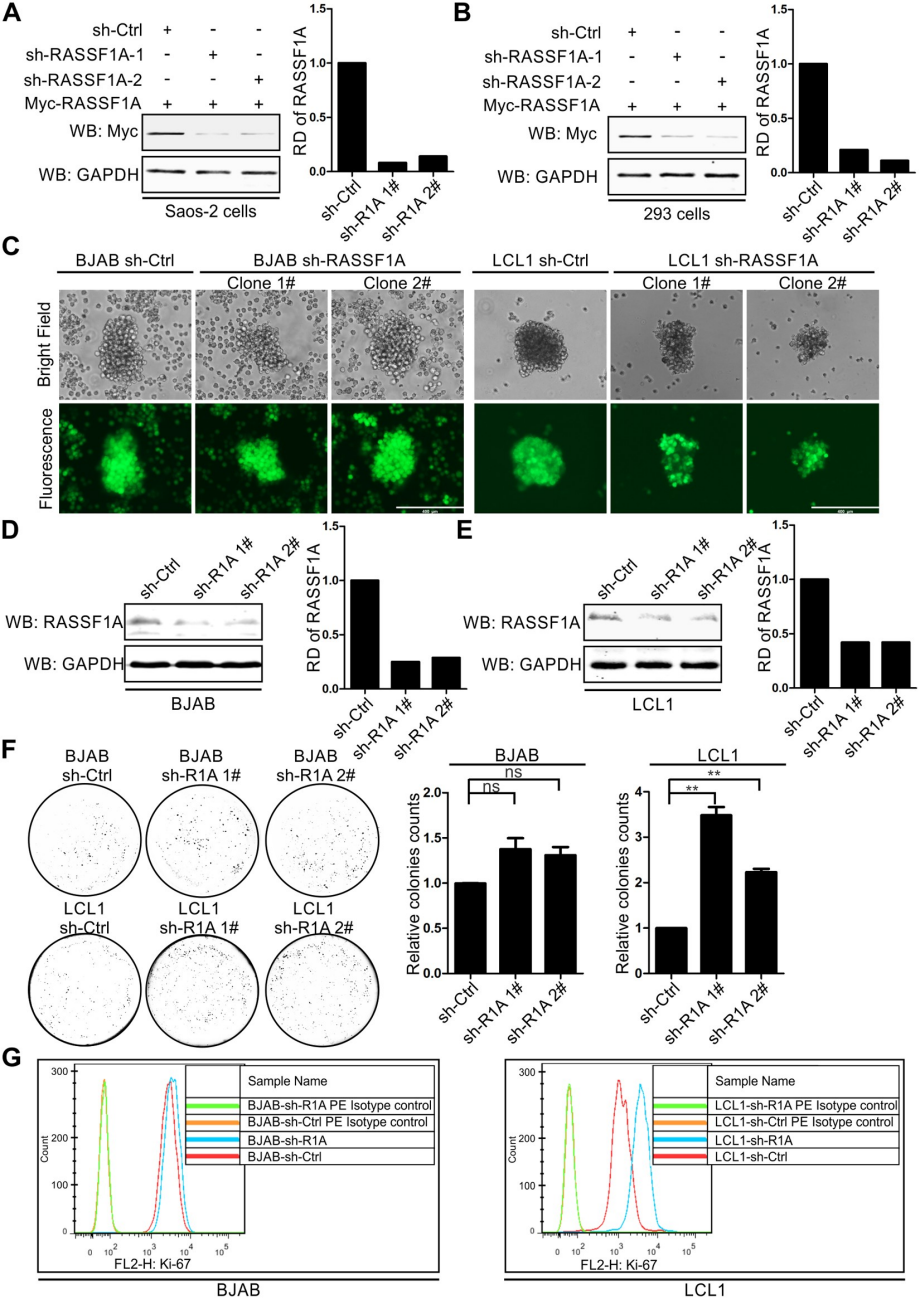
Knockdown of RASSF1A promotes transformation activity of LCL1. (A-B) The effect of sh-RASSF1A in reducing RASSF1A expression was detected in Saos-2 and 293 cells. A) Saos-2 and B) 293 cells were transfected with Myc-tagged RASSF1A and sh-Ctrl, or sh-RASSF1A-1 or sh-RASSF1A. 24 hours, the cells were harvested and the expression of RASSF1A was detected by western blot. (C-E) RASSF1A knocked down BJAB and LCL1 cells were constructed and detected. C) RASSF1A knocked down BJAB and LCL1 cells were constructed by lentiviruses and selected by puromycin for three weeks. GFP fluorescence was determined in the selected cells. D-E) The expression of endogenous RASSF1A was determined by western blot. (F) Colony formation was measured in RASSF1A knocked down BJAB and LCL1 cells by soft agar assays. The relative colony number was measured by Image J software. (G) Cell proliferation was monitored in RASSF1A knocked down BJAB and LCL1 cells. BJAB-sh-control, BJAB-sh-RASSF1A, LCL1-sh-control, and LCL1-sh-RASSF1A cells were harvested and incubated with 10 μl PE conjugated Mouse anti-Human anti-Ki-67 antibody or PE Mouse IgG1, κ Isotype control separately and analyzed by flow cytometry.

To evaluate the effect of EBNA3C on RASSF1A-regulated transformation activity, RASSF1A stably knocked-down BJAB and LCL1 cells were generated by lentivirus and selected using puromycin for 3 weeks (Fig 10A–10E). The ability of these selected cell lines to form colonies was determined by soft agar assays in RASSF1A knocked-down BJAB and LCL1 cells. The results showed that the colony formation was minimally enhanced in RASSF1A knocked-down BJAB cells. However, in RASSF1A knocked-down LCL1 cells, the relative colony number was dramatically increased (Fig 10F). Further, cell proliferation was monitored by flow cytometry using an antibody against Ki-67 in RASSF1A stably knocked-down BJAB and LCL1 cells. The results showed that knockdown of RASSF1A did not significantly increase cell proliferation in BJAB cells. However, cell proliferation was dramatically increased in RASSF1A stably knocked-down LCL1 cells (Fig 10G). These results demonstrated that EBNA3C promoted B-cell proliferation by suppressing RASSF1A expression.

### EBV-transformed LCLs promoted the G1-S transition by down-regulating RASSF1A-mediated inhibition of Cyclin D1 and Cyclin E

**Fig 11:**
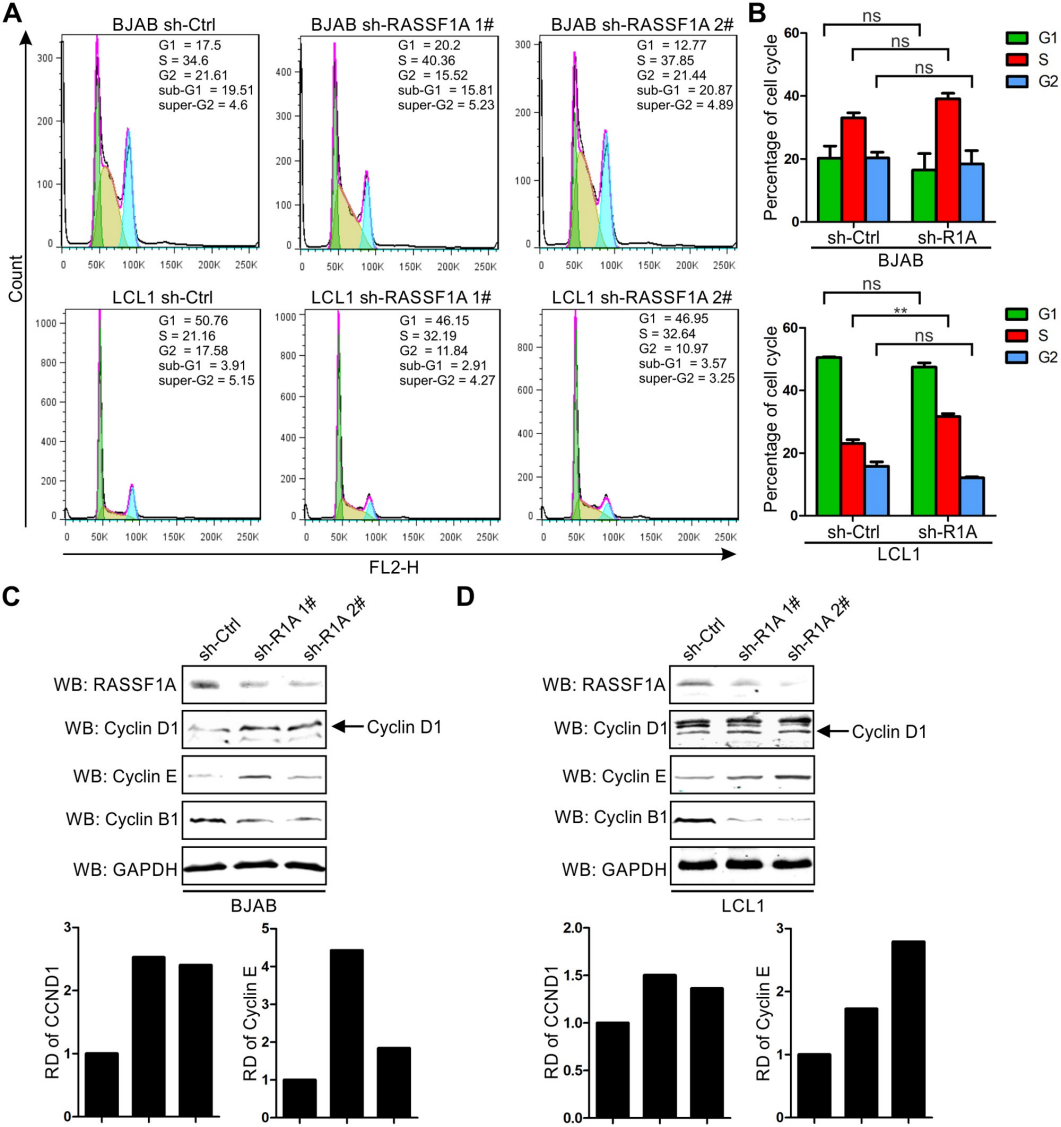
Knockdown of RASSF1A facilitates G1 to S transition by upregulating CyclinD1 and Cyclin E expression in LCL cells. A-B) RASSF1A knocked down BJAB and LCL1 cells were stained with PI staining buffer and analyzed by flow cytometry. Histogram showed the average values of two independent experiments. C-D) The expression of cyclin proteins were detected in RASSF1A knocked down BJAB and LCL1 cells. 10 million indicated cells were harvested and lysed for western blot. The expression of RASSF1A and cyclin proteins were detected by specific antibodies. The relative density (RD) of Cyclin D1 and Cyclin E were quantitated and shown.

Previous studies have shown that RASSF1A negatively regulates the G1 to S transition [80–82]. To further investigate the effect of EBNA3C on RASSF1A-mediated cell cycle arrest, cell cycle assays were performed in RASSF1A knocked-down BJAB and LCL1 cells and the percentages of cells in different phases were shown. The results showed that knockdown of RASSF1A in BJAB cells minimally increased the percentage of cells in the S phase. However, when RASSF1A was knocked down in LCL1 cells, the percentage of cells in the S phase was dramatically increased (Fig 11A and 11B). These results indicated that knockdown RASSF1A facilitated enhanced G1 to S transition in LCLs.

Previous studies reported that RASSF1A could block the cell cycle by inhibiting the accumulation of Cyclin D1 and Cyclin A2 [80, 83]. Furthermore, our lab showed that EBNA3C stabilizes and directly interacts with Cyclin D1 [25, 27], Cyclin A [29], and that EBNA3C plays an important role in G1-S transition by interacting with various Cyclin proteins. To further explore the mechanism employed by RASSF1A to modulate cell cycle activities, the expression of Cyclin D1, Cyclin E, and Cyclin B1 were analyzed in RASSF1A knocked-down BJAB and LCL1 cells. The results showed that Cyclin D1 and Cyclin E whose expression facilitated G1 to S transition were increased in RASSF1A knocked-down BJAB and LCL1 cells. However, Cyclin B1 whose expression facilitates G2 to M transition was significantly decreased (Fig 11C and 11D). These results suggested that EBNA3C regulated RASSF1A-mediated cell cycle arrest by modulating the expression of Cyclin D1 and Cyclin E. The downregulation of Cyclin B1 indicated that RASSF1A may also be implicated in mitotic transition.

### EBNA3C disrupted RASSF1A-mediated microtubule stability and induced chromosomal instability

**Fig 12:**
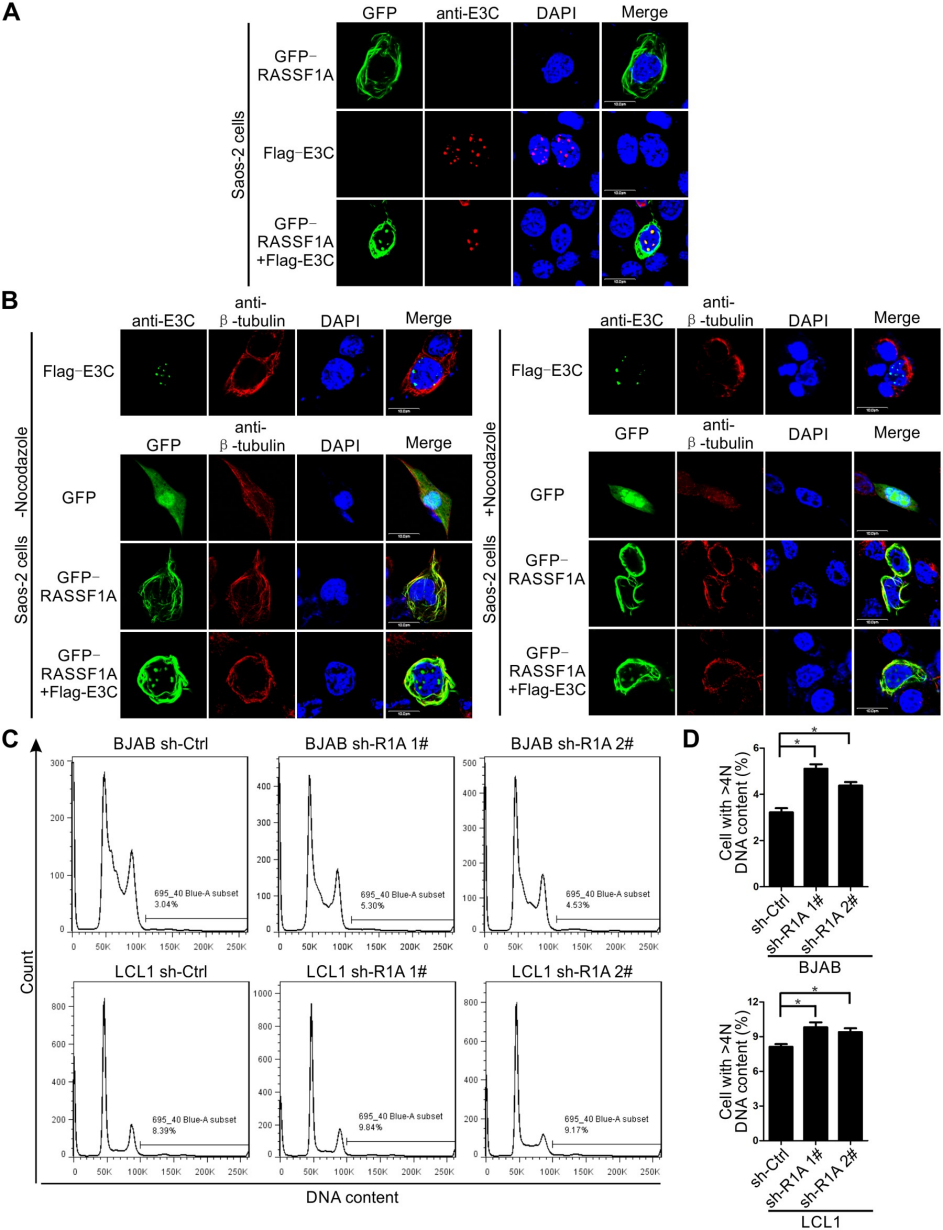
EBNA3C disrupts RASSF1A-mediated microtubule stability and induces chromosomal instability. A-B) EBNA3C decreased RASSF1A-mediated microtubule stability. A) Saos-2 cells were transfected with GFP-tagged RASSF1A and Flag-tagged EBNA3C. 24h post-transfection, the cells were harvested for immunofluorescence assays. B) Saos-2 cells were transfected with the indicated plasmids. 24h post-transfection, the cells were incubated with 10μM nocodazole for 1 hour. Then the cells were harvested for immunofluorescence assays to analyze microtubule stability. C-D) EBNA3C induced chromosomal instability in RASSF1A knocked down BJAB and LCL1 cells. 2 million RASSF1A knocked down BJAB and LCL1 cells were harvested and subjected to flow cytometry assay as described above. The DNA content in cells for G1, G2/M, and cells with increased DNA content was compared with cells in G2/M and was designated as 2N, 4N, and >4N. The cell percentage with DNA content >4N was calculated and shown. Histograms showed the average values of two independent experiments.

RASSF1A was reported to co-localize and promote the stabilization of microtubules [40]. The interaction between RASSF1A and microtubule plays a critical role in genomic stability and mitosis [54, 84, 85]. Since we demonstrated EBNA3C negatively regulated RASSF1A expression, it is likely that it would have an effect on microtubule stability. Immunofluorescence assays were performed to monitor microtubule stability using the inhibitor, nocodazole in treated or untreated Saos-2 cells. When GFP-tagged RASSF1A was expressed with EBNA3C, RASSF1A co-localized with discrete, punctate EBNA3C signals in the nucleus (Fig 12A). Next, we set out to detect the effects of EBNA3C on RASSF1A stabilized microtubules. In the absence of nocodazole, RASSF1A co-localized with filamentous microtubules when RASSF1A was expressed alone. In EBNA3C and RASSF1A co-expressed cells, a fraction of the RASSF1A signals were translocated into the nucleus and formed discrete, punctate structures similar to those observed above (Fig 12B). Interestingly, there was no obvious co-localization of RASSF1A and microtubules visualized in the nucleus even if some RASSF1A signal co-localized with microtubules in the cytoplasm. Importantly, when cells were treated with nocodazole, the negative control, GFP could not protect microtubules from depolymerization, and unorganized microtubules could be observed in the whole cell (Fig 12B). However, in RASSF1A expressed cells, organized filamentous microtubules still co-localized with RASSF1A in the cytoplasm even when the cells were treated with nocodazole (Fig 12B). This suggests that RASSF1A can play a role in enhancing microtubule stability. Furthermore, when RASSF1A was co-expressed with EBNA3C in nocodazole-treated cells, some discrete, punctate signals of RASSF1A was seen in the nucleus as expected. However, the organized filamentous microtubules were destroyed (Fig 12B). These results suggested that EBNA3C can destabilize RASSF1A-related microtubule stabilization.

Earlier reports have demonstrated that microtubule stability is important for RASSF1A-induced genomic stability [54, 84, 85]. Thus, it is expected that EBNA3C would likely affect chromosomal stability due to a decrease in RASSF1A-mediated microtubule stability. The DNA content of cells in G1 and G2/M with increased DNA content was compared with that in cells in G2/M designated as 2N, 4N, and >4N. Flow cytometry was performed to analyze the distribution of cells in different cell cycle phases in RASSF1A knocked-down BJAB and LCL1 cells and the DNA content was measured. When RASSF1A was knocked down, the percentage of the cell with DNA content >4N was increased compared with that in the sh-control group (Fig 12C and 12D). These results indicated that chromosomal aberrations were increased in RASSF1A knocked-down BJAB and LCL1 cells. These results suggest that EBNA3C disrupted RASSF1A-mediated microtubule stability inducing chromosomal instability.

## Discussion

**Fig 13:**
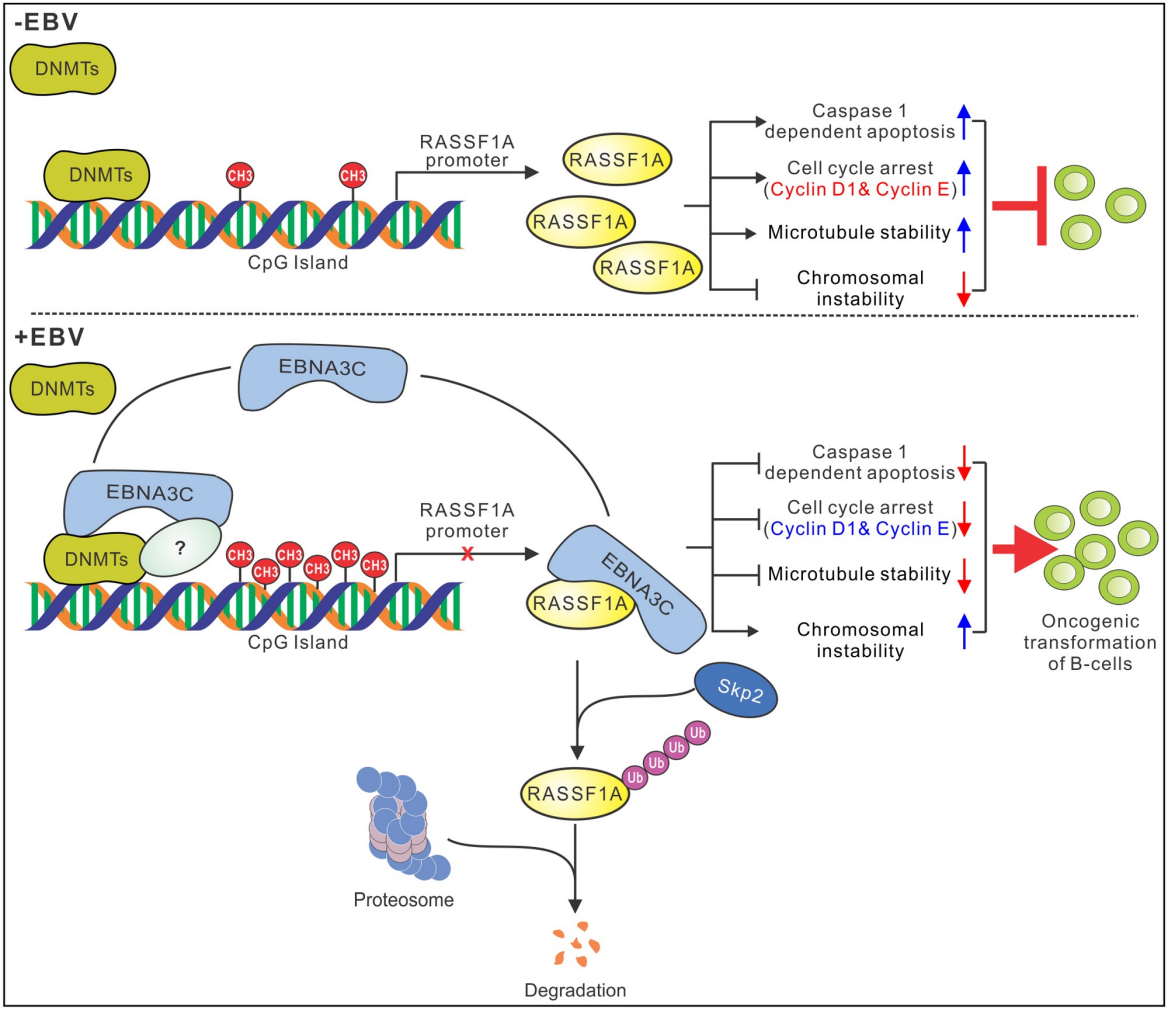
A schematic diagram shows the mechanism by which EBNA3C can suppress RASSF1A expression and promotes cell proliferation and transformation. EBAN3C directly interacts with RASSF1A and induces RASSF1A degradation via the ubiquitin-proteasome pathway. EBNA3C decreases RASSF1A promoter transcriptional activity by enhancing its methylation level, which is regulated by EBNA3C-induced DNMTs expression levels. EBNA3C inhibits RASSF1A-mediated cell apoptosis, disrupts RASSF1A-mediated microtubule and chromosomal stability and promotes cellular proliferation by upregulating Cyclin D1 and Cyclin E expression.

RASSF1A is a member of a family of tumor suppressors. It lacks any obvious enzymatic activity, but it regulates multiple biological processes by directly interacting with many cellular proteins, including p120^E4F^ [46], Cdc20 [44, 86], MST1 [48], and MAP1B [84]. Although RASSF1A serves as a scaffold protein for many signaling complexes, its level was reduced by promoter hypermethylation in a wide variety of human tumors [40]. RASSF1A promoter methylation has been seen in many EBV-related diseases, such as gastric cancer, nasopharyngeal carcinoma, and Hodgkin Lymphoma [39, 87]. The high inactivation frequency of RASSF1A in EBV related tumors suggests that it plays a pivotal role in EBV-related tumor development. However, the underlying mechanism of RASSF1A inactivation has not been fully explored. LMP1 was reported to regulate the expression of RASSF1A and to inhibit its transcription [54]. Numerous studies had demonstrated that down-regulation of RASSF1A is strongly associated with promoter hypermethylation in EBV associated cancers [29, 61]. Conflicting results were obtained with LMP1 in regulating DNMTs expression [53, 77]. LMP2A and EBNA3C were also shown to induce DNMT1 expression [55, 56]. However, the details as to the mechanism of RASSF1A inhibition is still not fully understood. Our results clearly demonstrated that EBNA3C specifically downregulated RASSF1A expression at the transcriptional and post-transcriptional levels (Fig 13).

Our data showed that EBNA3C specifically interacted with RASSF1A and suppressed RASSF1A expression through the ubiquitin-proteasome-dependent degradation pathway. Previous studies in our lab showed that EBNA3C could interact with several E3 ubiquitin-protein ligases, including SCF^Skp2^ and Mdm2 [58, 59]. Song MS, et al. reported that RASSF1A could directly interact with Mdm2, HAUSP, and Daxx protein [60]. Also, Skp2 was shown to regulate RASSF1A function by directly interacting with RASSF1A and mediating its degradation through the ubiquitin-mediated proteasome pathway [57]. In our study, we tested the effect of Skp2 mediated RASSF1A degradation. When Skp2 was co-expressed with EBNA3C, a significant decreased in RASSF1A expression was seen. However, the F-box deleted mutation of Skp2 failed to induce RASSF1A degradation. These results suggested that Skp2 was recruited by EBNA3C to regulate the degradation of RASSF1A. RASSF1A was also shown to regulate p53 degradation by Mdm2-mediated p53 ubiquitination. Conversely, p53 also induced RASSF1A suppression by recruiting DNMT1 and increasing the methylation levels of the RASSF1A promoter [61]. Additionally, p53 was also shown to interact with EBNA3C [24]. Therefore, we tested the possibility that EBNA3C repressed RASSF1A expression by a p53-mediated increase in DNMT1 expression. We tested the inhibition of EBNA3C on RASSF1A expression in several cell lines including Saos-2 cell, a p53 minus cell line, and p53 expressing cell lines, 293 and BJAB. RASSF1A expression was decreased to the same level in these three cell lines indicating that p53 is dispensable for EBNA3C-mediated RASSF1A down-regulation.

Transcriptional modification is another important strategy, which contributes to RASSF1A down-regulation. The evidence demonstrated that hypermethylation of the RASSF1A promoter can play a vital role in down-regulation of RASSF1A [40, 87, 88]. Our results suggested that RASSF1A mRNA expression was decreased by EBNA3C, and that EBNA3C inhibited the transcription activity of RASSF1A promoter by enhancing the methylation level of RASSF1A promoter. A previous study by Young Seo et al. showed that LMP1 activated DNMT1, DNMT3a and DNMT3b expression in NPC cells [53]. However, another study in Hodgkin’s lymphoma by Leonard et al. showed that LMP1 downregulated the expression of DNMT1 and DNMT3b, but upregulated DNMT3a expression [77]. LMP2A also increased the methylation of PTEN in gastric cancer cell lines by inducing DNMT1 expression [55]. Earlier studies in our lab showed that DNMT3A and DNMT3B, but not DNMT1, gradually increased in EBV primary infected-B cells [37]. Furthermore, studies in our lab showed that EBNA3C not only increased the expression of DNMT1 but also interacted with DNMT1 in EBNA3C-expressing cell lines [56]. In this study, we confirmed that DNMT1 was slightly increased in EBNA3C stably expressing BJAB7 and BJAB10 cell lines. However, the expression of DNMT1 and DNMT3b was decreased in EBV-transformed LCL cell lines, as well as EBV positive BL41-B95.8 cell line. Our results are consistent with the results of Leonard et al [77]. The expression of DNMT3a was increased in EBNA3C positive and EBV positive cell lines, but we failed to detect the association between EBNA3C and DNMT3a or DNMT3b. These results suggested that other cellular proteins are likely mediating the interaction between EBNA3C and DNMTs. Additional efforts will elucidate the mediator, which bridges the interaction between EBNA3C and DNMTs. Although DNMT1 expression was decreased in EBV positive cell lines, we cannot rule out the possibility that EBNA3C-mediated DNMT1 plays a role in modifying RASSF1A promoter methylation levels. In EBV positive cell lines, the expression of DNMT1 is regulated by several viral proteins. DNMT1 expression was decreased in the cell, but for a specific gene, the methylation levels still can be enhanced by DNMT1. The same situation may apply to EBNA3C-mediated RASSF1A promoter hypermethylation. However, additional experiments are needed to fully understand this interplay.

RASSF1A plays an important role in apoptosis, cell cycle arrest, and microtubule and chromosome stability [40]. To explore the underlying mechanism for EBV-induced lymphomagenesis, the effect of EBNA3C on RASSF1A-related cell functions were evaluated. From our results, we know that RASSF1A induces apoptosis through the caspase dependent apoptotic pathway. EBNA3C can antagonize RASSF1A-mediated cell apoptosis and facilitates cell proliferation by regulating the levels of multiple Cyclin proteins. Previous reports have shown that the accumulation of Cyclin D1 and Cyclin A2 was inhibited in RASSF1A expressing cells [80, 89]. In our study, we found that other cyclin proteins are also involved in RASSF1A-mediated cell cycle arrest. In RASSF1A knocked-down LCL1 cell lines, the expression of Cyclin D1 was increased slightly. However, Cyclin E expression was strongly increased. Cyclin E is a Cyclin member that determines the initiation of DNA replication and is indispensable for G1 to S transition [90, 91]. Our cell cycle experiments showed an increase in the percentage of cell in the S phase, and Cyclin E promotes Cyclin A expression by phosphorylating p27^Kip1^ with the help of Cdk2 [90]. Thus, even if we did not detect the expression of Cyclin A2, it is reasonable to speculate that knocking down RASSF1A would improve Cyclin A expression levels to facilitate the G1 to S transition. Furthermore, Cyclin B1 involved in mitosis [92, 93] was dramatically decreased in RASSF1A knocked down BJAB and LCL1 cells. A decrease in the percentage of cells in G2 phase for BJAB sh-RASSF1A and LCL1 sh-RASSF1A was observed in our cell cycle experiments. These results suggested that RASSF1A not only modulated G1 to S transition but can also regulate mitosis.

Previous studies reported that RASSF1A localized to cytoplasmic microtubules during interphase, localized to the centrosome in prophase, and localized to spindle microtubules and spindle poles during metaphase and anaphase [40]. When RASSF1A Ser202/Ser203 was mutated to Glu202/Glu203, it would disrupt the interaction between RASSF1A and microtubules and so abolish its ability to induce M-phase arrest [85]. These results indicate that microtubule stability and interaction were critical for RASSF1A to regulate mitotic arrest. When cells were treated with nocodazole, RASSF1A protected microtubule from depolymerization. However, EBNA3C attenuated RASSF1A function on microtubule stability. RASSF1A failed to protect microtubule from depolymerization in presence of EBNA3C in cells treated with nocodazole. A higher percentage of cells with >4N DNA content was observed in RASSF1A knocked down BJAB and LCL1 cells when compared with the BJAB sh-Ctrl and LCL1 sh-Ctrl cells. These results indicated that knocking down RASSF1A induced chromosomal abnormalities and reduced genomic stability in these cells. Ultimately, knocking down RASSF1A contributed to an increase in cell proliferation and transformation in LCLs. However, the details of the RASSF1A-mediated regulatory network are yet to be fully unveiled in EBV-transformed LCLs. Further detailed investigation in RASSF1A knock-out mice will provide evidence of the biological function of RASSF1A in EBV-associated tumorigenesis.

In summary, our findings now demonstrate a role for EBNA3C in regulating the activities of the tumor suppressor RASSF1A and have enhanced our understanding of the many roles of EBNA3C in EBV-infected B cells. These studies now reveal another layer to the mechanisms employed by EBNA3C in suppressing the function of RASSF1A to promote EBV-mediated transformation. Furthermore, it provides another valuable target for rationale antiviral strategies and the development of new drugs to combat the associated pathologies.

## Materials and methods

### Cells and antibodies

RASSF1A antibody (eB114-10H1) used for western blot and Co-immunoprecipitation was purchased from Thermo Fisher. RASSF1A antibody (3F3) used for immunofluorescence was obtained from Santa Cruz Biotechnology, Inc. (Santa Cruz, CA). DNMT1, DNMT3a, and DNMT3b antibodies were purchased from Abcam (ab87656, ab13888, ab13604, Cambridge, UK). Mouse monoclonal antibodies against PARP1 (F-2) and GFP (B-2) were obtained from Santa Cruz Biotechnology, Inc. Mouse monoclonal antibodies against Flag antibody (M2) was bought from Sigma-Aldrich (St. Louis, MO, USA). Mouse anti Myc (9E10), mouse anti-EBNA3C (A10) [94], and mouse anti-HA (12CA5) were prepared in our lab from the hybridoma. Rabbit polyclonal antibody specific for EBNA3C was obtained from Cocalico Biologicals, Inc. (Reamstown, PA) and has been described previously [95]. Other antibodies to GAPDH, caspase-1, caspase-1 P20, caspase 3, caspase 7, caspase-7 p20, Skp2 p45 Antibody (A-2), and Cyclin proteins were purchased from Santa Cruz biotechnology, Inc.

Saos-2 (human osteosarcoma cell line) and HEK-293 (human embryonic kidney cell line) were provided by Jon Aster (Brigham and Woman’s Hospital, Boston, MA). Saos-2 and HEK-293 were maintained in Dulbecco’s modified Eagle’s medium (DMEM; Gibco) supplemented with 5% Bovine Growth Serum (BGS, HyClone Bovine Growth Serum). BJAB, BL41, and BL41-B95.8 cell lines were kindly provided by Elliott Kieff (Harvard Medical School, Boston, MA). The EBV-negative and positive cell lines, Sav I and Sav III cell lines, Mutu I and Mutu III cell lines were kindly provided by Dr. Paul M. Lieberman (The Wistar Institute, Philadelphia, PA).). EBV-negative Akata cells and B95-8 EBV infected Akata cells (Akata-EBV) stably expressing type III latent proteins were kept in our lab and previously described [15]. BJAB stably expressing EBNA3C cells (BJAB7, BJAB10) were prepared by transfecting pZipneo-EBNA3C into BJAB cells and selected with neomycin [96]. LCL1 and LCL2 cells were EBV-transformed immortalized LCLs (lymphoblastoid cell line) generated in our laboratory [30]. LCL1-sh-EBNA3C and LCL1-sh-ctrl were generated in our laboratory and previously described [27]. BJAB, BJAB7, BJAB10, LCL1, LCL2, EBV negative and positive cell lines, LCL1-sh-EBNA3C and LCL1-sh-ctrl were cultured in RPMI 1640 media (Gibco) with 7% BGS. BJAB-sh-RASSF1A and LCL1-sh-RASSF1A were generated in our laboratory and maintained in RPMI 1640 media containing 7% FBS with 1 μg/ml puromycin.

### Plasmid constructs

The plasmids pA3F-EBNA3C, pA3F-EBNA3C 1–365, pA3F-EBNA3C 366–620 and pA3F-EBNA3C 621–992 encoding full-length Flag-tagged EBNA3C and truncated mutations of EBNA3C have been described previously [65]. The full-length RASSF1A was amplified from total RNA by reverse transcription PCR via using primers 5’-CGCGGATCCATGTC-GGGGGAGCCTGAG-3’ and 5’-CCGCTCGAGACTCCCCAGAGTCATTTTCCTTCAGG-3’. The PCR products were cloned into pA3M and pEGFP-N1, respectively. The pA3M-RASSF1A cDNA was used as the template for PCR amplification to generate RASSF1A mutants. The PCR products were cloned into pA3M. pGL4.2-R1A-promoter used to express RASSF1A promoter was amplified from genomic DNA by primers: 5’-GAAGATCTAGCCCAGGGTGACAGAGCCA-AATG-3’ and 5’-CCAAGCTTGGCCCGGTTGGGCCCGTGCTTC-3’. PCR products were inserted into pGL4.20[luc2/Puro] vector (Promega Corporation, Madison, USA) by Bgl II and Hind III.

#### Transfection

BJAB cells were transfected by electroporation with a Bio-Rad Gene Pulser II electroporator. 10 million BJAB cells in exponential phase were harvested by centrifugation at 800 rpm for 5 minutes. The pellet was washed once with 15 ml PBS and resuspended with 0.4 ml cold RPMI medium without serum, mixed with plasmids and cells. The mixture was transferred into a 0.4-cm cuvette and placed on ice for 5 minutes. Electroporation was performed at 220 V and 975 μF. After electroporation, the cuvettes were placed on ice for another 10 minutes and the cells were transferred into pre-warmed RPMI medium and incubated at 37°C. 24 hours post-transfection, the transfection efficiency was monitored. 48 hours post-transfection, the cells were harvested and lysed for western blot.

#### RNA isolation and real-time PCR

10 million B-cells were harvested and the total RNA was extracted by using Trizol reagent (Invitrogen, Inc., Carlsbad, CA). Then cDNA was generated using the Superscript II reverse transcriptase kit (Invitrogen, Inc., Carlsbad, CA) according to the manufacturer’s protocol. Quantitative Real-time PCR analysis was performed by using SYBR green Real-time master mix (MJ Reserch Inc., Waltham, MA). The results were normalized to the endogenous control, GAPDH. Primers for GAPDH were 5’-TGCACCACCAACTGCTTAG-3’ and 5’-GATGCAGG-GATGATGTTC-3’ [27]. Primers for RASSF1A were 5’-ACAGCAACCTCTTCATGAGCT-3’ and 5’-CAAGGAGG-GTGGCTTCTTGCT-3’.

### Co-immunoprecipitation

Transfected cells or 60 million B-cells were harvested and washed with ice-cold PBS twice at the indicated time points. The cells were lysed with 400μl ice-cold RIPA buffer [1% Nonidet P-40 (NP-40), 10 mM Tris (pH8.0), 2 mM EDTA, 150 mM NaCl, supplemented with protease inhibitors (1 mM phenylmethylsulphonyl fluoride (PMSF), 1 μg/ml each aprotinin, pepstatin and leupeptin] for 1 hour. The lysates were centrifuged at 15000 rpm for 10 minutes. 5 μl were taken out for protein concentration measurement (Bio-Rad Protein Assay Dye Reagent Concentrate). 5% of the lysates were boiled with SDS loading buffer and used as input. 3μg total protein was used for Co-IP for B cells. Lysates were precleared by mixing with normal control serum and 20 μl of a 2:1 mixture of Protein-A/G Sepharose beads (GE Healthcare Biosciences, Pittsburgh, PA) for 2 hours at 4°C with rotation. The precleared supernatants were incubated with 1 μg of specific antibody and 30 μl of a 2:1 mixture of Protein-A/G Sepharose beads overnight at 4°C with rotation. Beads were collected by centrifugation at 4,000 rpm for 2 min. The supernatants were discarded and the beads extensively washed with RIPA buffer 3 times. The beads were boiled with SDS protein loading buffer. The input and the bound proteins were analyzed by SDS-PAGE and detected by Western blotting using specific antibodies. Images were visualized on a Licor Odyssey imager (LiCor Inc., Lincoln, NE). The relative density (RD) of the bands was analyzed and quantified Image Quant software from Licor Odyssey imager.

### Confocal microscopy

Immunofluorescence assays were performed as described earlier with minor modifications [97]. Saos-2 cells in 12-well plates were grown on coverslips and transfected with indicated plasmids. 24 h post-transfection, cells were washed with ice-cold PBS three times and fixed with 4% paraformaldehyde. 2 million B-cells were harvested and washed with PBS once and air-dried. 4% paraformaldehyde was used to fix the cells at room temperature for 20 minutes. Then the fixed cells were washed with PBS and permeabilized with 0.2% Triton X-100 for 20 min at room temperature. After blocking by 3% bovine serum albumin for 0.5 hours at room temperature, the cells were incubated with rabbit anti-EBNA3C antibody and/ or mouse anti RASSF1A or mouse anti-Myc or mouse anti-β-tubulin overnight at 4 °C. The cells were washed by PBS for three times and incubated with the Alexa Fluor 594 goat anti-rabbit (Thermo; 1:100) or Alexa Fluor 488 goat anti-mouse (Thermo; 1:1000) secondary antibody for 1 h at room temperature. The cells were stained with DAPI (4’, 6-diamidino-2-phenylindole) for 10 minutes. After being washed with PBS three times, the coverslips were turned over and put on a glass slide with a drop of mounting media. Confocal images were collected by Fluoview FV300 confocal microscope.

### Stability assay

Saos-2 cells were transfected with Myc-tagged RASSF1A with Flag-tagged EBNA3C or Flag-tagged vector by calcium phosphate. 24 hour after transfection, cells were treated with 40 μg/ml cycloheximide (CHX; CalBiochem, Gibbstown, NJ) and harvested at specific time points. 10 million B-cells were incubated with 40 μg/ml CHX and harvested every two hours. The cells were lysed and the lysates were subjected to western blot with specific antibodies. Band intensities were quantitated by Image Quant software from Licor Odyssey imager.

### Dual-luciferase reporter assay

The dual-luciferase reporter assays were performed as described earlier with minor modifications [15]. Saos-2 and HEK293 cells in 12-well plate were co-transfected with pGL4.2-R1A-promoter, pRL-TK (Promega, Madison, WI, USA) and increasing amounts of Flag-tagged EBNA3C. Total amounts of plasmid were kept constant by co-transfecting with the vector. 48 hours post-transfection, cells were harvested and lysed in 150 μl lysis buffer. 20 μl lysates were used for dual-luciferase reporter assay according to the manufacturer’s instructions (Promega, Madison, WI, USA). The expression of Flag-EBNA3C was detected by western blot. All assays were repeated at least three times for reproducibility.

### Genomic DNA extraction

Genomic DNA extraction was performed as previously described with minor modification [98]. 5 million cells were harvested and washed once with PBS. Cells were resuspended with 500 μl HMW buffer (10mM Tris-HCl pH 8.0, 150mM NaCl, 10mM EDTA, 0.5% SDS) at 55 °C for 2 hours. The cells lysates were mixed with proteinase K to a final concentration of 0.5 μg/ml and incubated at 37 °C overnight. Genomic DNA was extracted by phenol/chloroform/isopropanol.

### Methylation-specific polymerase chain reaction (MSP)

MSP was performed as previously described [75]. 1 μg Genomic DNA was subjected to bisulfite conversion and purification by using the EZ DNA Methylation Gold Kit (Zymo Research Corporation, CA, USA) following the manufacturer’s instructions. 100 ng and 50 ng bisulfite-modified DNA was used in 15 μl methylation-specific PCR (MSP) reaction to amplify methylated (M) and unmethylated (U) DNA fragments. The primers used for RASSF1A Methylation-specific PCR are listed below RASSF1A-U-F: 5’-GGGGTTTGTTTTGTGGTT-TTGTTT-3’ and RASSF1A-U-R: 5’-AACATAACCCAATTAAACCCATACTTCA-3’; RASSF1A-M-F: 5’-GGGTTCGTTTTGTGGTTTCGTTC-3’ and RASSF1A-M-R: 5’-TAACCC-GATTAAACCCGTACTTCG-3’ [75]. The primers used for c-Myc Methylation-specific PCR are as follow c-Myc-U-F: 5’-GGGATTTTTGATTAA-AGTGTGGT-3’ and c-Myc-U-R: 5’-CCCTT-TCTCTACTACTCCTCCATAA-3’; c-Myc-M-F: 5’-GACTCTTGATCAAAGCGCGGC-3’ and c-Myc-M-R: 5’-CTTTCTCTGCTGCTCCTCCGTAG-3’. The optimized thermal profile included initial denaturing at 95°C for 5 minutes, followed by 35 cycles with a 30s denaturation step at 94°C, 45s of annealing at 50°C, 1minute of extension at 72°C and a final extension at 72°C for 10 minutes. PCR products were analyzed by agarose gel electrophoresis and ethidium bromide staining and quantitated by Image J software.

### Lentiviral production and infection

sh-RNAs targeted RASSF1A and sh-Skp2 were constructed by annealing two pairs of primers. The primers for RASSF1A shRNAs are 5’-tcgagtgctgttgacagtgagcgaGCTTGAACAAGGACG-GTTCTTtagtgaagccacagatgtaAAGAACCGTCCTTGTTCAAGCgtgcctactgcctcggaa–3’ (shRASSF1A-1), and 5’- tcgagtgctgttgacagtgagcgaCGGTTCTTACACAGGCTTCATtagtgaagcc-acagatgtaATGAAGCCTGTGTAAGAACCGgtgcctactgcctcggaa–3’ (sh-RASSF1A-2). The primers for Skp2 shRNAs are 5’-tcgagtgctgttg-acagtgagcgaGATAGTGTCATGCTAAAGAAT-tagtgaagccacagatgtaATTCTTTAGCATGACACTATCgtgcctactgcctcggaa-3’ and 5’- cgcgttccga-ggcagtaggcacGATAGTGTCATGCTAAAGAATtacatctgtggcttcactaATTCTTTAGCATGACACTATCtcgctcactgtcaacagcac-3’. The sense stranded oligos were annealed with their respective anti-sense stranded oligos, and then cloned into Xho I and Mlu I digested pGIPZ vector. A negative control was constructed by the same method with another pair of primers. The sense strand is 5’-TCTCGCTTGGGCGAGAGTAAG–3’ (Dharmacon Research, Chicago, IL). Lentivirus production and transduction have been described previously [15, 65].

### Colony formation assay

Saos-2 cells and HEK293 in 6-well plate were transfected with GFP, control vector, Myc-RASSF1A, Myc-RASSF1A-E4F1 alone or together with Flag-EBNA3C. The transfected cells were selected in DMEM with 2mg/ml G418 (Sigma-Aldrich, St. Louis, MO, USA). Two weeks later, 4% paraformaldehyde was used to fix the cell colonies at room temperature for 30 minutes and stained with 0.1% crystal violet for 0.5 h. The colonies were scanned by BioRAD ChemiDOC MP Imaging system and the relative colony number was measured by Image J software. All assays were repeated three times for reproducibility.

### Soft agar assays

The soft agar assays were performed in RASSF1A knocked down BJAB or LCL1 cells. 1 ml 1% agar in supplemented with RPMI media was poured into 6-well plate and set aside to solidify. 1×10^5^ cells were mixed with 1 ml 0.3% agar/medium and poured on the top of the 1% agar layer. Two weeks later, colonies were stained with 0.1% crystal violet for 30 minutes and scanned by BioRAD ChemiDOC MP Imaging system. The relative colony numbers were measured by Image J software. All assays were repeated three times for reproducibility.

### Flow cytometry

Flow cytometry was performed with RASSF1A knocked down BJAB or LCL1 cells to detect the cell cycle and DNA content. 1 million stable cells were collected and suspended with 300 μl PBS containing 2% BGS. Then the cells were fixed with 1ml ice-cold 100% ethanol for 24 hours at 4°C. After been washed with PBS containing 2% BGS once, the cells were stained with PI staining buffer (0.5 mg/ml propidium iodide in PBS, 50 μg/ml RNase A) for 30 minutes at room temperature and analyzed using a FACS Calibur (BD LSR II Special Order System, USA) and FlowJo software (Treestar, Inc., San Carlos, CA).

### CSFE and Ki-67 proliferation assay

HEK293 in 6-well plate were transfected with control vector, Myc-RASSF1A, Myc-RASSF1A-E4F1 alone or together with Flag-EBNA3C. Have been selected G418 for two weeks, 1x10^6^ cells were incubated with PBS containing 2% BGS and 2.5 μM CFSE for 10 minutes in 37 °C. 1ml cold DMEM was added to the cells to stop the staining. The cells were washed with DMEM twice and then suspended with 0.4 ml DMEM. 1x10^5^ cells were seeded and incubated in 37 °C incubator for 3 days. The cells were harvested and fixed with 100% cold ethanol for flow cytometry assay.

1x10^7^ BJAB-sh-control, BJAB-sh-RASSF1A, LCL1-sh-control, and LCL1-sh-RASSF1A cells were harvested and fixed with 5 ml 80% ethanol for 24 hours in -20 °C. The cells were washed with PBS containing 2% BGS twice and 100 μl (1x10^6^) cells were mixed with 10 μl PE conjugated Mouse anti-Human anti-Ki-67 antibody or PE Mouse IgG1, κ Isotype control (BD Biosciences) separately at room temperature for 30 minutes in the dark. After being washed once, the cells were suspended with 0.5 ml PBS containing 2% BGS and analyzed by flow cytometry.

### Statistical analysis

All experiments were repeated at least three times for accuracy. Mean scores with standard deviation (SD) are represented. The student’s t-test was used to calculate the significance of differences in the mean scores in our study. A P-value below 0.05 was considered to be statistically significant (*P < 0.05; **P < 0.01; ***P < 0.001; NS, not significant).
